# The effects of locus coeruleus ablation on mouse brain volume and microstructure evaluated by high-field MRI

**DOI:** 10.3389/fncel.2024.1498133

**Published:** 2024-12-11

**Authors:** Rasmus West Knopper, Christian Stald Skoven, Simon Fristed Eskildsen, Leif Østergaard, Brian Hansen

**Affiliations:** ^1^Center of Functionally Integrative Neuroscience, Department of Clinical Medicine, Aarhus University, Aarhus, Denmark; ^2^Sino-Danish Center for Education and Research, University of Chinese Academy of Sciences, Beijing, China

**Keywords:** locus coeruleus, DSP-4, DKI, volumetrics, mouse brain, behavior

## Abstract

The locus coeruleus (LC) produces most of the brain’s noradrenaline (NA). Among its many roles, NA is often said to be neuroprotective and important for brain upkeep. For this reason, loss of LC integrity is thought to impact brain volume and microstructure as well as plasticity broadly. LC dysfunction is also a suspected driver in the development of neurodegenerative diseases. Nevertheless, the impact of LC dysfunction on the gross structure and microstructure of normal brains is not well-studied. We employed high-field ex vivo magnetic resonance imaging (MRI) to investigate brain volumetrics and microstructure in control (CON) mice and mice with LC ablation (LCA) at two ages, representing the developing brain and the fully matured brain. These whole-brain methods are known to be capable of detecting subtle morphological changes and brain microstructural remodeling. We found mice behavior consistent with histologically confirmed LC ablation. However, MRI showed no difference between CON and LCA groups with regard to brain size, relative regional volumes, or regional microstructural indices. Our findings suggest that LC-NA is not needed for postnatal brain maturation and growth in mice. Nor is it required for maintenance in the normal adult mouse brain, as no atrophy or microstructural aberration is detected after weeks of LC dysfunction. This adds clarity to the often-encountered notion that LC-NA is important for brain “trophic support” as it shows that such effects are likely most relevant to mechanisms related to brain plasticity and neuroprotection in the (pre)diseased brain.

## 1 Introduction

The locus coeruleus (LC) is a small nucleus located in the pons. Acting as the principal source of noradrenaline (NA) in the central nervous system (Szabadi, 2013), it is associated with various cognitive functions, including attention (Janitzky et al., 2015; Lapiz and Morilak, 2006; Spencer and Berridge, 2019), arousal (Berridge et al., 2012), and stress response (Valentino and Van Bockstaele, 2008). Furthermore, a potential trophic influence of the LC on brain structure has been of interest at least since the review by Foote et al. (1983) and often encountered in the literature when listing the functions of LC (Edeline et al., 2011; Marien et al., 2004; Poe et al., 2020). LC’s role in this area is often described using the blanket term “trophic support” which covers a variety of mechanisms. More specifically, studies suggest LC-NA to influence neurodevelopment (Fauser et al., 2020), -plasticity (Hagena et al., 2016), -protection (Yao et al., 2015), and maintenance (Giorgi et al., 2020). Nevertheless, the literature does not draw a clear picture of the role of the LC in these processes.

Initial studies of LC’s involvement in neurodevelopment hinted an influence on the early postnatal ontogenesis of the neocortex in rodents (Maeda et al., 1974) and the synaptogenesis in later developmental stages (Sanders, 2016). However, other studies using oxidopamine (6-OHDA) in newborn rats found no effect on the development of the visual cortex (Ebersole et al., 1981) nor the neocortex (Wendlandt et al., 1977). Conversely, increased synaptic density in the rat visual cortex after neonatal 6-OHDA treatment has been reported Parnavelas and Blue (1982), indicating a suppressive effect of NA on synaptogenesis. In line with this, NA appears to inhibit cell proliferation and differentiation within the periventricular regions during brain development (Fauser et al., 2020) through β-adrenergic receptor signaling (Weselek et al., 2020). However, NA also has the ability to promote neurogenesis in the hippocampal subgranular zone (Weselek et al., 2020). Therefore, existing studies disagree about NA’s role, potentially indicative of regional and age-related variations in NA influence.

In the mature brain, neuroplasticity, e.g., in the context of learning, is improved by optogenetic excitation of the LC (Glennon et al., 2023). Multiple NA-related mechanisms likely support learning. Examples include LC-induced reconfiguration of functional connectivity toward the salience network (Zerbi et al., 2019), astrocytic integration of salient stimuli mediated by α_1_-adrenergic receptors (Rupprecht et al., 2024), long-term potentiation (LTP) and long-term depression (LTD)-mediated synaptic plasticity from β-adrenergic receptors activation (Adamsky et al., 2018; Hagena et al., 2016), and hippocampal neurogenesis through β-adrenergic receptors (Weselek et al., 2020). Thus, the LC not only plays a role in the early stages of the developing brain but continues to support the brain’s adaptation to new stimuli (i.e., plasticity) throughout life.

The LC-NA system also plays a complex role in neuroprotection. NA is anti-inflammatory by suppressing microglial cytokine production while increasing the breakdown of neurotoxins, which induces microglial migration and phagocytosis mediated through β-adrenergic receptors (Heneka et al., 2010). Recent studies show that NE signaling through β-adrenergic receptors in the awake brain inhibits microglial surveillance, dynamically modulating microglial interaction with neurons and synaptic plasticity, thereby suggesting a critical regulatory role of NE in microglial function under different states of arousal (Liu et al., 2019; Stowell et al., 2019). Additional neuroprotective effects of the LC-NA system are mediated through the co-release of brain-derived neurotrophic factor (BDNF) binding to the TrkB receptor (Liu et al., 2015). NA is also implicated in brain maintenance through the regulation of brain blood flow (Bekar et al., 2012) and vascular permeability (Raichle et al., 1975) by acting on pericytes and astrocytes (Giorgi et al., 2020), thus affecting metabolic supply. However, suppression of NA has also been found to ameliorate glymphatic drainage and inflammatory response after traumatic brain injuries (Hussain et al., 2023), suggesting a complex role of the LC-NA system, influencing multiple aspects of brain function as well as both brain energetic supply and waste removal. Notably, LC pathology seems to precede other symptoms of Alzheimer’s and Parkinson’s disease (Braak and Del Tredici, 2011; Vermeiren and De Deyn, 2017), raising the question of whether LC dysfunction is a driving force in the disease development or an early, parallel process in the already diseased brain.

The role of LC in brain trophic support is multifaceted and even conflicting under different circumstances. The literature on this topic is currently ambiguous and a clear role for NA in the support and protection of brain structures cannot be assigned. Despite the advances since the review by Foote et al. (1983), the understanding of how long-term LC dysfunction impacts structure and microstructural composition in the otherwise normal brain remains lacking. As outlined above, LC ablation leads to various alterations in the brain. How this manifests itself in the brain structure has, however, mostly been investigated by histology. Our study seeks to contribute to this area by doing whole-brain comparisons of brains from controls and LC-ablated mice in two age groups: one where the noradrenergic system is still maturing (LCA13) and one where it is fully developed (see Discussion). In both groups, LC ablation is achieved using the neurotoxin N-(2-Chloroethyl)-N-ethyl-2-bromobenzylamine hydrochloride (DSP-4). We perform *ex vivo* high-field MRI to assess brain volumetrics and sensitive indices of brain microstructure in these groups.

## 2 Materials and methods

### 2.1 Animals

All animal procedures were conducted in accordance with the ARRIVE guidelines (Percie Du Sert et al., 2020) and the European Council Directive (2010/63/EU) and approved by the Animal Experiments Inspectorate (permit no: 2020-15-0201-00684) in Denmark. The mice were housed in individually ventilated cages (GR900, Tecniplast, Italy) containing hiding structures, bedding materials, and chewing sticks with water and food *ad libitum* and kept under a 12-h light/dark cycle (lights on: 07:00 h) at 23 ± 1°C room temperature and 54 ± 2% air humidity.

**FIGURE 1:**
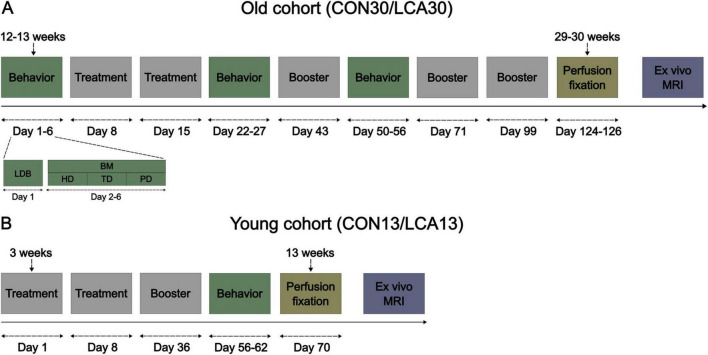
The experimental timeline of the two cohorts of mice. **(A)** The old cohort of mice where the brain tissue was perfusion fixed at 29–30 weeks of age. The days count under the event blocks are days relative to the first treatment. The behavior event consists of two behavioral tests, detailed under days 1–6. The mice underwent a light-dark box (LDB) test on day 1 and a Barnes maze (BM) test on day 2–6, divided into habituation day (HD), training day (TD), and probe day (PD). The mice received a booster injection every 4 weeks after the initial two treatments. **(B)** The young cohort of mice, perfusion fixed 13 weeks of age, was weaned 3 weeks after birth and received treatment immediately after.

The study included 35 male C57BL/6NRj mice (Janvier, France) divided into two age groups. The first cohort of mice (*n* = 20; 10 weeks old) was housed in four cages of five, earmarked, and randomly assigned to the control group (CON; *n* = 10) or the LC ablation group (LCA; *n* = 10) within each cage. Subsequently, the animals underwent one pre-treatment and two post-treatment test batteries consisting of the light-dark box (LDB) test and the Barnes maze (BM) test (see Figure 1 for details). The brains were then fixed and harvested for in-skull *ex vivo* MRI. For the second cohort, five timed pregnant females (Janvier, France; 7 weeks old) arrived at the vivarium on gestation day 8 or 14 and gave birth on gestation day 20. All 15 male pups were weaned at 3 weeks of age and divided into four cages comprised of mice from one or two litters, earmarked, and assigned either the CON (*n* = 7) or the LCA (*n* = 8) group within each cage. For convenience, the groups are annotated CON13/LCA13 and CON30/LCA30, referring to the age at euthanasia (13 and 30 weeks of age). The second cohort only underwent one post-treatment test battery followed by the same *ex vivo* MRI protocol as the first cohort (Figure 1). All behavior tests were conducted by the same experimenter. Consequently, both cohorts were divided in two and separated by approximately 1 week to ensure the tests were conducted within the same time window of the day for every animal.

### 2.2 Locus coeruleus ablation

To induce LC ablation, we used the procedure described in our previous study (Markussen et al., 2023), corresponding to the LCA2 group. This procedure was found to produce reliable ablations and induce behavioral changes consistent with our understanding of LC function. Briefly, the LCA groups were treated with the LC-selective (Ross and Stenfors, 2015) neurotoxin DSP-4 (Sigma-Aldrich, product no.: C8417) dissolved in sterile phosphate-buffered saline (PBS) immediately before intraperitoneal injection (50 mg/kg). The CON group was correspondingly injected with PBS. Two injections, separated by 1 week, were initially administered. Hereafter, monthly booster injections were administered to suppress compensatory mechanisms (Fritschy and Grzanna, 1992). Body weight and animal behavior were monitored throughout the study. One mouse (LCA30) unexpectedly died 1 week after its third booster injection, with no abnormality in behavior or body weight observed.

### 2.3 Behavioral tests

For comparison with our previous results (Markussen et al., 2023) and for further characterization of the LC-ablated mouse model, the mice underwent a behavioral test battery consisting of the light-dark box (LDB) test and the Barnes maze (BM) test. The animals were placed in customized acclimatization boxes inside a room equipped with an automated video tracking system and software for behavior analysis (Noldus EthoVision XT 17) 30 min before the tests. The CON30/LCA30 groups underwent one pre-treatment and two post-treatment test batteries. A provisional data check suggested a risk of the mice being too adapted to the tests and, thereby, blurring the ablation effect. Consequently, the test batteries were reduced to a single post-treatment test in the CON13/LCA13 groups. We outline the behavior test procedures briefly below. In the analysis of behavioral data, recorded trials of outliers were inspected for faulty tracking and manually corrected if necessary. Detailed descriptions of the procedures, experimental setup, and data analysis from these two behavior tests were provided previously (Markussen et al., 2023).

#### 2.3.1 Light-dark box test

We use the LDB test to examine the effect of LC ablation on the anxiety of open, illuminated environments and the novelty-induced exploratory behavior (Kulesskaya and Voikar, 2014). The light-dark box (Noldus, Wageningen, Netherlands) consists of a two-compartment box with 1/3 being covered and dark and 2/3 being open and illuminated with approximately 120 lx. The mice were initially placed in the light compartment, facing toward the walls, furthest away from the entrance to the dark compartment. The test was 10 min in duration and was divided into two time bins of 5 min during the analysis to uncover habituation effects. Indicators of anxiety-like behavior (Hascoët, 1998; Simon et al., 1994) extracted from the test included (1) time spent in the light compartment, (2) the percentage of time in the light compartment along the walls, (3) the number of transitions from light to dark compartment, (4) latency to the enter the dark compartment, and (5) latency to enter the light compartment after entering the dark compartment. Furthermore, motility metrics (Recober et al., 2010) were computed to ensure that no motor-related deficiencies were affecting the behavioral tests. The data were not subjected to inferential statistics but served as a control for behavioral differences. The motility metrics included (1) the mean velocity for each compartment, (2) the mean velocity during movement only (when the mouse moved faster than 2 cm/s), and (3) the percentage of time spent moving in a compartment. See Supplementary Figure 1 for an example of the LDB test arena.

#### 2.3.2 Barnes maze test

The BM test was used to assess how spatial learning (Gawel et al., 2019) was affected by the ablation. The BM table (Ugo Basile, Gemonio, Italy) was a one-meter-in-diameter circular table with 20 evenly distributed holes along the table perimeter. The BM test consisted of three phases, distributed over 5 days: (1) One habituation day, including 3 min of exploring the table without an escape box and visual cues, (2) three training days (TDs) with three trials per mouse per day, each trial lasting until the mouse enters the escape box or for a maximum of 3 min, and, (3) one probe day (PD) with a duration of 3 min without the escape box. In each trial, the mouse was positioned in the center of the table. Spatial learning was probed using four metrics: (1) Time before escape, (2) percentage of time spent in the entry zone, (3) number of visits to non-target zones, and (4) latency to entry zone. Differences in search strategies between the groups were assessed by registering the number of different non-target visits and the percentage of time in each table quadrant. The TDs and PDs were separated in the analysis. See Supplementary Figure 1 for an example of the BM test arena.

### 2.4 Magnetic resonance imaging

#### 2.4.1 Sample preparation

Before perfusion fixation of the brains, the mice were anesthetized using 5% isoflurane mixed with a flow of 0.2 L/min oxygen and 0.2 L/min air, followed by an intraperitoneal injection of 0.5 mL pentobarbital (400 mg/ml, Alfasan, Woerden, Netherlands). After thoracotomy, the brains were perfusion fixed using heparinized (Heparin, 0.2 mL/100 mL, 5000 IU/mL, LEO, Denmark, PN: 464327) PBS (pH = 7.3, Sigma-Aldrich, USA, PN: P4417) for 2–3 min until the liver appeared pale, followed by a buffered formaldehyde solution (4%, pH = 7.0, PN: 9713.1000) for 3 min. After decapitation, the mandible and extracranial tissue were removed from the skull to avoid susceptibility artifacts from air bubbles trapped in fur and cavities during imaging. Hereafter, the samples were stored in the formaldehyde solution for at least 4 weeks. Before imaging, the samples were washed in PBS for at least 24 h on a rocker to increase signal by removal of excess fixative (Shepherd et al., 2009). As a standard procedure (Bay et al., 2018; Chuhutin et al., 2020; Jespersen et al., 2010; Khan et al., 2016; Vestergaard-Poulsen et al., 2011), the samples were subsequently mounted in a 15 mL centrifuge tube filled with a perfluorocarbon-based liquid (Fluorinert, 3M, PN: FC-770). This in-skull brain preparation ensures that brain shape is unaffected during imaging as in Ardalan et al. (2022) and Qvist et al. (2018), enabling atlas-based segmentation as in Lindhardt et al. (2023).

#### 2.4.2 MRI data collection

MRI was performed using a 9.4 T preclinical system (BioSpec 94/20, Bruker Biospin, Ettlingen, Germany) equipped with a bore-mounted 25 mm quadrature transmit-receive coil. To reduce sample vibrations, the tube containing the sample was fitted into the coil using a custom polyethylene foam cylinder. In-house 3D-printed sample holders ensured consistent positioning of the samples throughout the experiments. High-resolution *B*_0_ shimming using Bruker’s MAPSHIM was performed to improve echo-planar imaging (EPI) employed here for DKI acquisition. Both DKI data and structural data were acquired for each sample.

DKI data was collected using an 8-segmented diffusion-weighted spin-echo EPI sequence with a 150 × 150 μm in-plane resolution and 60 slices with a thickness of 250 μm. Initially, five unweighted images were acquired for normalization of the 30 isotopically distributed encoding directions at each of the three non-zero b-values (0.5, 1.0, 2.0 ms/μm^2^). Additional scan parameters were time between diffusion gradients (Δ) = 15 ms, duration of diffusion gradients (δ) = 6 ms, 20 averages, effective echo time (TE) = 27.7 ms, repetition time (TR) = 3500 ms, bandwidth = 278 kHz, with a scan time of 19 h 26 m 40 s.

Two structural data sets were acquired per sample. A rapid acquisition with relaxation enhancement (RARE) sequence with a 50 × 50 μm in-plane resolution, 250 μm slice thickness, and 60 slices in total was used for multi-atlas segmentation (MAS, details below) for ROI-specific extraction of DKI parameters. These data had the same slice thickness and positions as the DKI data described above to increase segmentation fidelity of the DKI data. The scan parameters used were effective TE = 10.5 ms, TR = 3000 ms, 30 averages, and RARE factor = 2, constituting a scan time of 2 h 28 m 30 s. For the volumetric analysis, we used a 3D fast low-angle shot (FLASH) sequence to obtain data with an isotropic resolution of 50 μm. Scan parameters were: TR = 88.5 ms, TE = 13.7 ms, matrix size = 360 × 198 × 300, FOV = 18 × 9.9 × 15 mm, and 4 averages, resulting in a scan time of 6 h 31 m 21 s.

#### 2.4.3 Multi-atlas segmentation and manual LC delineation

To systematically extract regional information from the mouse brain, a multi-atlas segmentation (MAS) (Ma et al., 2014, 2019) was performed using 10 *ex vivo* NeAt templates from C57/BL6J mice (Ma et al., 2005, 2008). This was done similarly to our previous study (Lindhardt et al., 2023) with minor modifications. Briefly, the MAS pipeline was utilized for the structural RARE images, having the same slice package as the DKI sequence. N4 bias field correction proved beneficial due to some sensitivity drop in the outer edges of the coil’s axial FOV. The non-linear transformation step, as per default recommendation in the pipeline (Ma et al., 2014, 2019), was, therefore, also carried out. This procedure resulted in high-resolution labeled images, downsampled to match the in-plane resolution of the diffusion MRI, to extract DKI metrics in anatomically well-defined regions of interest (ROIs). See Supplementary Figure 2 for a segmentation example.

Microstructural effect of ablation in the LC-containing part of pons was assessed. For this, ROIs were manually drawn on the high resolution structural scans for each using a mouse brain atlas as guide (Paxinos and Franklin, 2001). Care was taken to ensure as small ROIs as possible to avoid contamination by partial volume effects. These ROIs were then downsampled to match the DKI resolution and used to extract pixel-wise values of MD and MK for all animals. See Supplementary Figure 3 for these segmentations. Pixelwise metric values were then pooled by groups for analysis.

#### 2.4.4 Volumetric analysis

After quality checking the MRI scans, three subjects (2 × CON30 and 1 × LCA13) were excluded from the volumetric analysis. For the remaining samples, the high-resolution FLASH images were processed using an in-house pipeline applying B1 inhomogeneity correction (Sled et al., 1998), denoising (Coupe et al., 2008), and intensity normalization. Spatial alignment with a high-resolution template of the C57BL/6J mouse (Dorr et al., 2008) was done by manually initializing a linear registration (Collins et al., 1994) followed by a non-linear registration (Avants et al., 2014). Neuroanatomical labels from the C57BL/6J mouse atlas were subsequently transformed and resampled to scanner native space using the calculated deformation fields and affine transformations for calculation of individual regional volumes. All ROI volumes were normalized to the total brain volume (TBV).

Cortical thickness was calculated as previously described (Lerch et al., 2008). Briefly, Laplace’s equation, with a fixed boundary condition that differed at the inner and outer surfaces, was solved using the inner and outer surfaces of the cortex defined on the anatomical atlas and transformed to the given mouse. For each point on the cortical surface, the length of a streamline connecting the inside and outside surfaces was used to define the thickness. Cortical thickness was averaged within the bilateral frontal, occipital, and parieto-temporal lobes as well as the entorhinal cortex.

#### 2.4.5 Diffusion kurtosis analysis

Before analysis, all DKI data were initially inspected visually for quality, resulting in the exclusion of four subjects (2 × CON30, 1 × LCA13, and 1 × LCA30) from the DKI analysis due to artifacts. DKI data from the remaining samples were preprocessed in MATLAB (MathWorks Inc., v. 2022a), including Rician noise floor correction (Koay and Basser, 2006), denoising (Veraart et al., 2016), and correction for Gibbs ringing (Kellner et al., 2016). After preprocessing, DKI data analysis was performed using in-house MATLAB scripts as previously described in Ardalan et al. (2022) and Hansen et al. (2017) yielding metrics of mean water diffusivity (MD), tissue anisotropy (FA) and indices of tissue microstructure (tensor-based mean kurtosis, MK) in each voxel (see Hansen et al. (2017) for details and review). Before the DKI analysis, ROI voxel values four times the median absolute deviation were filtered out. The ROIs were bilaterally pooled before the statistical analysis.

### 2.5 Statistical analysis

Volumetric and diffusion metrics were probed using a mixed permutation analysis of variance (ANOVA). Behavioral outcomes were analyzed using mixed permutation ANOVAs and bootstrapped Welch’s t-tests. Statistically significant interaction effects were further decomposed into simple main effects permutation analyses. Finally, bootstrapped Welch’s t-tests were conducted for *post hoc* testing, reporting the bootstrapped confidence interval (CI) of the group difference and bootstrapped p-values, which were then corrected for multiple comparisons using the Holm method. TBV-ROI correlations were assessed using Pearson correlation with bootstrapped CI. Both resampling methods used 10000 repetitions. Pooled values of MD and MK from the LC-containing region of pons were tested for difference using a permutation test with 100000 repetitions. Effect size maps of cortical thickness differences are provided as Cohen’s d. The p-maps are based on a two-tailed t-test at each vertex on the cortical surface. The statistical analysis was carried out in R using the packages “permuco” (Frossard and Renaud, 2021), “wPerm” (Weiss, 2015), “MKinfer” (Kohl, 2024), and “confintr” (Mayer, 2023) and MATLAB using the function “PermutationTest” (Omran, 2024). Figures were made in Matlab or Python using the library “matplotlib” (Hunter, 2007).

## 3 Results

### 3.1 Animal well-being

Supplementary Figure 4 shows the progression of body weight measurements between the CON and LCA groups. In general, we did not see any signs of lowered well-being of the animals during the study.

### 3.2 Behavioral results

Initially, behavioral tests were conducted on the CON30/LCA30 groups.

#### 3.2.1 Light-dark box

**FIGURE 2:**
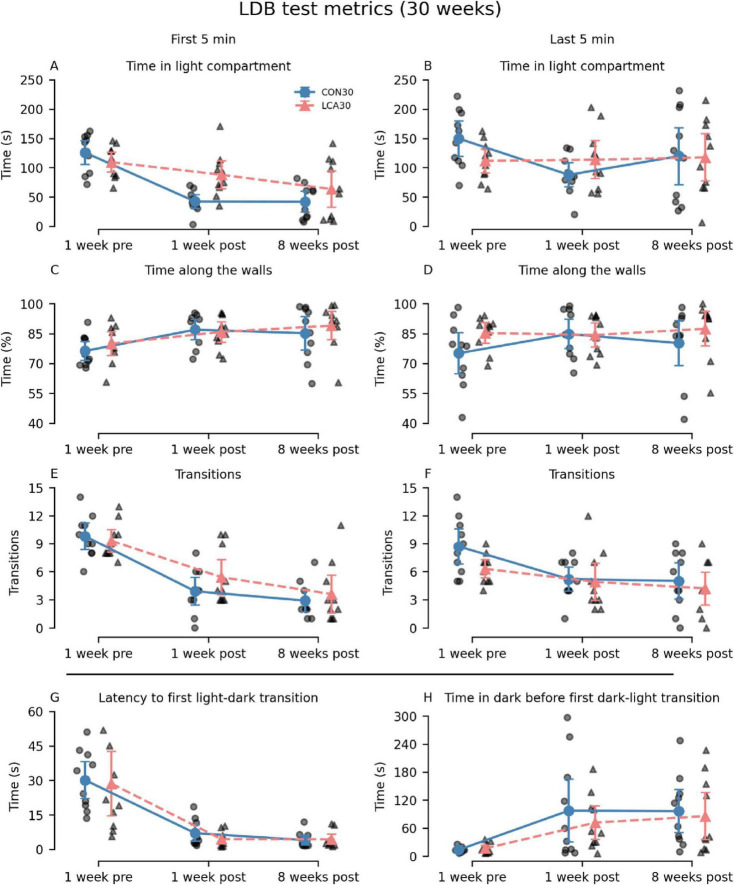
Light-dark box (LDB) test results of the CON30/LCA30 groups. The LDB test metrics were separated into time bins of 5 min at three time points relative to the treatment (the “time” factor in the statistical analysis). Note that the columns show time bins (first and last 5 min) except for the two last subplots where this division was not applicable. The x-axis shows the time of the experiment relative to the treatment. **(A,B)** A pronounced difference was observed in the time spent in the light compartment 1 week after treatment, where the LCA30 group stayed longer in the light compared to the CON30 group. **(C,D)** No difference was observed in the time along the walls. **(E,F)** The LCA30 group had more transitions between compartments 1 week after the treatment during the first 5 min but did not differ at other time points. **(G)** The groups did not differ in the latency to the first light-to-dark transition. **(H)** The LCA30 returned faster to the light compartment after the first transition to the dark compartment. See Supplementary Tables 1, 2 for detailed descriptive statistics. The group mean with 95% CI is plotted next to the individual observations.

Overall, the LCA30 group exhibited an increased duration spent in the light compartment compared to the CON30 group, particularly evident in the 1-week post-treatment test (Figures 2A, B), where the time was increased by 108 and 29% during the first and last 5 min, respectively. Here, the LCA30 group also exhibited a 38% increase in the number of transitions during the first 5 min (Figure 2E). Additionally, the LCA30 group showed a 26% reduction in time spent in the dark compartment before returning to the light after the initial transition between compartments (Figure 2H). Beyond these metrics and time points, the groups remained comparable, as detailed in Figure 2. See Supplementary Tables 1, 2 for descriptive group statistics. For the time spent in the light compartment (Figures 2A, B), the three-way mixed permutation ANOVA revealed a significant treatment × time interaction (*F*(2,36) = 4.44, *p* = 0.020) along with main effects observed for time bins (*F*(1,18) = 10.00, *p* = 0.006) and time (*F*(1,18) = 9.40, *p* = 0.000). A *post hoc* test of the significant main effects analyses revealed a treatment group difference across time bins 1 week before the LC ablation, where the LCA30 group spent 20% less time in the light compartment (*p* = 0.020, 95% CI [−6, −49]). This was opposite 1 week after the ablation, where LCA30 spent 55% more time in the light compared to CON30 (*p* = 0.014, 95% CI [11, 61]). For the time along the walls (Figures 2C, D), only the time term was found to be significant (*F*(2,36) = 4.59, *p* = 0.016), while the remaining terms were not. The number of transitions (Figures 2E, F) differed between the LDB tests (*F*(2,36) = 35.96, *p* < 0.000), as did the time bin × time interaction (*F*(2,36) = 4.67, *p* = 0.019). However, no significant treatment-related differences were observed, including the treatment × time bin × time interaction (*F*(2,36) = 0.02, *p* = 0.982). A two-way mixed permutation ANOVA revealed a significant main effect of time for the duration spent in light before the first transition (*F*(2,36) = 33.12, *p* < 0.000) (Figure 2G), while no significant main effect of treatment (*F*(1,18) = 0.18, *p* = 0.685) nor the treatment × time interaction (*F*(2,36) = 0.09, *p* = 0.920) was observed. Comparable results were seen in the time in dark before returning to light (Figure 2H) with a significant main effect of time (*F*(2,36) = 8.25, *p* = 0.002), while neither the treatment term (*F*(1,18) = 0.36, *p* = 0.550) nor the treatment × time interaction (*F*(2,36) = 0.24, *p* = 0.793) were significant. Motor-related metrics can be found in Supplementary Figures 5, 6. Here, a small difference was observed in mean velocity and percentage of time spent moving in the light compartment 1 week after treatment.

**FIGURE 3:**
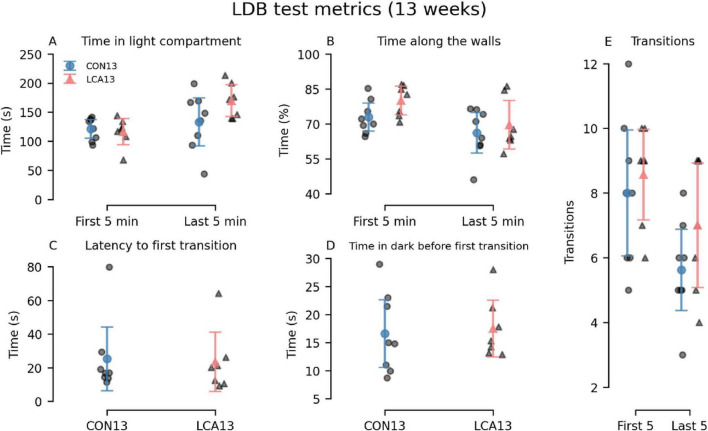
Light-dark box (LDB) test results of the CON13/LCA13 groups divided into 5 min time bins. **(A)** Notably, the LCA13 group spent more time in the light compartment during the last 5 min. The groups did not differ in the **(B)** time along the walls, **(C)** latency to first light-to-dark transitions, and **(D)** time in the dark before the first transition to light. **(E)** The number of transitions was higher during both time bins. The group mean with 95% CI is plotted based on individual observations. See Supplementary Tables 3, 4 for descriptive statistics.

The differences between the LCA13 and CON13 groups were comparable with the older cohort. The LCA13 group spent more time in the light compartment during the last 5 min, alongside elevated percentages of time along the walls and transitions throughout both time intervals compared to the CON13 group. See Figure 3 for an overview. The two-way mixed permutation ANOVA showed a significant difference in time spent in the light compartment (Figure 3A) between the time bins (*F*(1,13) = 5.21, *p* = 0.041). However, neither the treatment term (*F*(1,13) = 2.64, *p* = 0.125) nor treatment × time bin interaction (*F*(1,13) = 2.10, *p* = 0.170) were significant. Similar results were obtained for time along the walls, where no treatment effects were observed (Figure 3B). The two time bins differed significantly from each other (F(1,13) = 10.59, *p* = 0.008), while neither treatment (*F*(1,13) = 1.85, *p* = 0.200) nor the treatment × time bin interaction (F(1,13) = 0.50, *p* = 0.480) showed statistical significance. Similarly, the difference in the number of transitions (Figure 3E) between the two time bins was significant (*F*(1,13) = 7.16, *p* = 0.020), but not for the treatment term (*F*(1,13) = 2.25, *p* = 0.156) and the treatment × time bin interaction (*F*(1,13) = 0.30, *p* = 0.597). Bootstrapped Welch’s t-tests did not reveal significant differences between the groups for both latency to first light to dark transition (*p* = 0.811, 95% CI [−17.47, 19.58]) (Figure 3C) and the time in dark before returning to the light compartment (*p* = 0.787, 95% CI [−7.24, 5.35]) (Figure 3D). No motor-related differences were observed between the treatment groups (Supplementary Figure 7).

#### 3.2.2 Barnes maze

**FIGURE 4:**
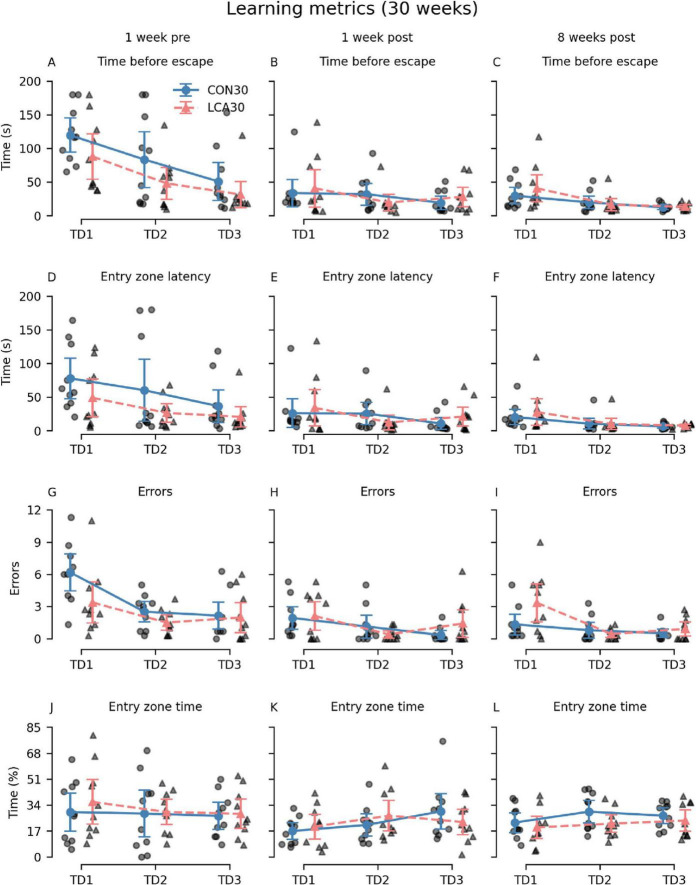
The Barnes maze (BM) learning metrics of the CON30/LCA30 groups on the three training days (TDs) at each of the three time points. **(A–C)** The groups did not differ in time before entering the escape box. **(D–F)** No differences were observed between the groups in the latency to enter the zone around the escape box. **(G–I)** The LCA30 group had more erroneous visits to non-target entry zones 8 weeks after the treatment compared to the CON30 group and even their baseline performance 1 week before the treatment. **(J–L)** The groups were not different in the time spent in the entry zone. The group means with 95% CI are plotted as error bars next to the individual observations. See Supplementary Tables 5–8 for descriptive statistics.

Generally, the LCA30 group performed better than the CON30 group during the initial TDs in most of the learning metrics before the groups received their treatment (Figures 4A, D, G, J). However, these differences largely vanished in the two post-treatment tests except for the error count 8 weeks after treatment. Here, the LCA30 group exhibited more errors during the initial TD compared to the CON30 group (Figure 4I). Notably, the learning curve across TDs was comparable with the pre-treatment learning curve, with an even higher number of initial errors on TD1 (Figure 4G). The three-way mixed permutation ANOVA of the error count found a significant treatment × time × day interaction (*F*(4,36) = 3.59, *p* = 0.015), corresponding to the data of Figures 4G–I). However, a simple interaction effect analysis of the treatment × time interaction at all levels of the time factor resulted in no significant outcomes. No treatment differences were observed for the time before escape (Figures 4A–C), latency to first visit in entry zone (Figures 4D–F), and time in entry zone (Figures 4J–L). See Supplementary Tables 5–8 for descriptive group statistics.

**FIGURE 5:**
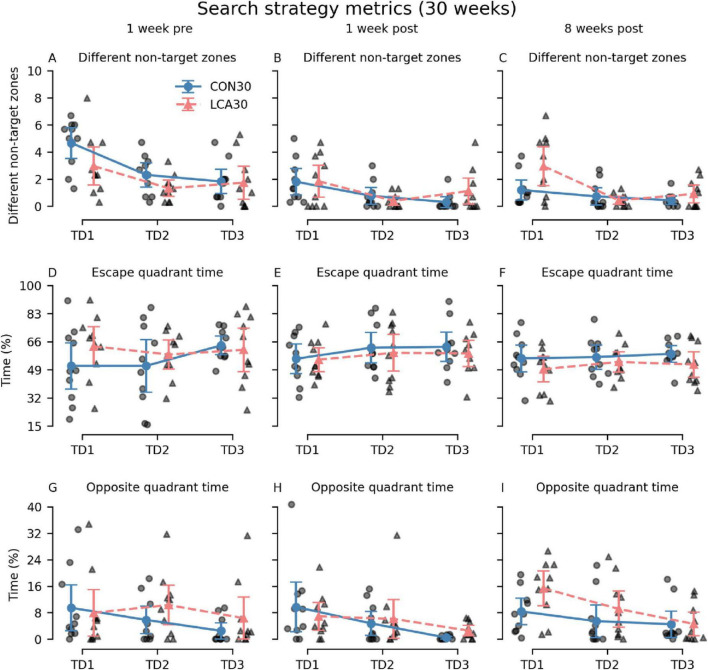
The Barnes maze (BM) search metrics of the CON30/LCA30 groups on the three training days (TDs) at each of the three time points. **(A–C)** The LCA30 groups had more visits to non-target zones 8 weeks after the treatment compared to the CON30 group. **(D–F)** No differences were observed between the groups in the time spent in the quadrant of the table containing the escape box. **(G–I)** The LCA30 group spent more time in the quadrant of the table opposite the escape box 8 weeks after the treatment. The group means with 95% CI are plotted as error bars next to the individual observations. See Supplementary Tables 5–8 for descriptive statistics.

Additional metrics were analyzed to investigate whether differences in search strategy could mask effects of the ablation (Figure 5). Interestingly, 8 weeks after the treatment, we observed that the LCA30 group had a 142% increase in visits to different non-target zones on TD1 compared to the CON30 group (Figure 5C) while also spending 83% more time in the quadrant opposite to the escape box, despite performing better during the pre-treatment test (Figures 5G–I). The three-way permutation ANOVA of the different non-target zones found a significant treatment × time interaction (*F*(2,18) = 3.61, *p* = 0.047). However, the simple main effect analysis showed no difference. See Supplementary Tables 5–8 for descriptive group statistics. No significant differences were observed between the treatment groups on the PD (Supplementary Figure 8).

In general, no differences were observed between the LCA13 and CON13 in any of the BM metrics during the TDs. As expected, both groups exhibited the ability to learn, and a significant main effect of day was found in all metrics. See Supplementary Figure 9 for an overview. No differences were observed between LCA13 and CON13 on the PD (Supplementary Figure 10).

### 3.3 MRI results

#### 3.3.1 Ablation effect in the subregion of pons containing the LC

**FIGURE 6:**
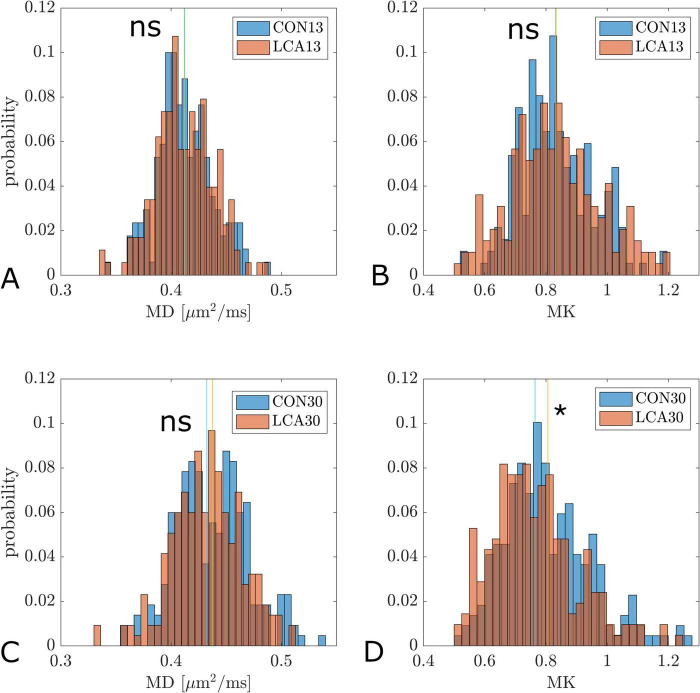
Histograms of pixel-wise MD and MK values in the LC-containing part of pons from all animals pooled by group. **(A)** Distribution of MD values in CON13 and LCA13 (ns). **(B)** Distribution of MK values in CON13 and LCA13 (ns). **(C)** Distribution of MD values in CON30 and LCA30 (ns). **(D)** Distribution of MK values in CON30 and LCA30 (*p* = 0.002). In all panels vertical lines represent distribution mean values. Significance was determined by permutation test by the mean (100000 permutations). ns, not significant. *p < 0.05.

Figure 6 shows histograms of pixel-wise MD and MK values in the LC-containing part of pons from all animals pooled by group. MD is not significantly different between LCA and CON in either age group (Figures 6A–C). MK is not significantly different between LCA13 and CON13 although the mode of the LCA13 distribution (0.52) is lower than in the CON13 group (0.54) and the distribution variances were significantly different (two-sided *F*-test, *p* = 0.007). MK is significantly decreased in the LCA30 compared to CON30 (*p* = 0.002) (Figure 6D). Here, the group mean for LCA30 is 0.76 ± 0.14 compared to 0.81 ± 0.14 for CON30. In each plot, vertical lines mark the mean value for each group. Reported values are mean ± SD.

#### 3.3.2 Volume

**FIGURE 7:**
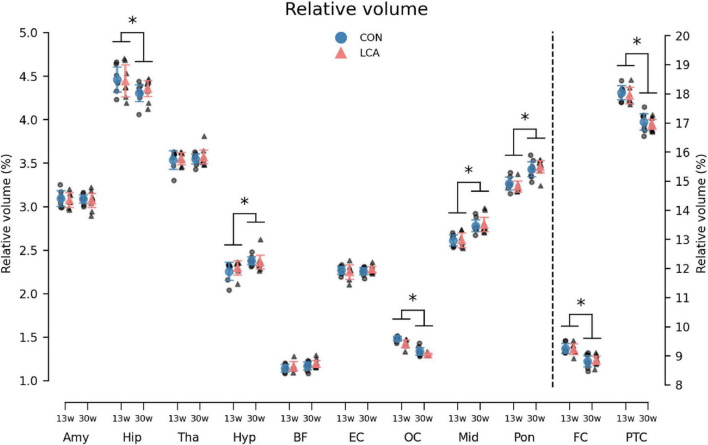
The volume of the 11 regions of interest (ROIs) given as the percentage of total brain volume (TBV). Note that FC and PTC belong to the right axis. Notably, the LCA and CON groups of the same age were similar, while a difference was observed between the age groups. The group mean with 95% CI is superimposed on the individual observations. See Supplementary Table 9 for detailed descriptive statistics. 13w, 13 weeks old; 30w, 30 weeks old; Amy, amygdala; hip, hippocampus; tha, thalamus; hyp, hypothalamus; BF, basal forebrain; EC, entorhinal cortex; OC, occipital cortex; mid, midbrain; pon, pons; FC, frontal cortex; PTC, parieto-temporal cortex. *Age difference (*p* < 0.05).

Overall, the volume of all ROIs in the LCA groups did not differ from the CON groups, regardless of their age. Instead, the age groups differed in multiple ROIs (Figure 7). The three-way mixed permutation ANOVA found a significant age × ROI interaction (*F*(10,270) = 53.96, *p* < 0.000) and two main effects (age: *F*(1,27) = 33,83, *p* < 0.000, ROI: *F*(10,270) = 42560,00, *p* < 0.000). No significant main effect of treatment (*F*(1,27) = 0.15, *p* = 0.711), treatment × age interaction (*F*(1,27) = 0.40, *p* = 0.541), or treatment × age × ROI interaction (*F*(10,270) = 0.15, *p* = 0.999) were observed. Post hoc tests of the significant simple main effects analysis showed age-related differences in the hippocampus (*p* = 0.029, 95% CI [0.02, 0.22]), hypothalamus (*p* = 0.003, 95% CI [−0.16, −0.04]), occipital cortex (*p* < 0.000, 95% CI [0.10, 0.16]), midbrain (*p* < 0.000, 95% CI [−0.23, −0.12]), pons (*p* < 0.000, 95% CI [−0.25, −0.14]), frontal cortex (*p* < 0.000, 95% CI [0.28, 0.55]), and parieto-temporal cortex (*p* < 0.000, 95% CI [0.80, 1.18]). See Supplementary Table 9 for descriptive group statistics.

Although the ROI volumes remained consistent between treatments, the TBV, from which the relative ROI volumes were derived, conversely differed across treatment and age (Supplementary Figure 11A). While the LCA13 group had smaller TBV compared to the CON13 group, this pattern was reversed among the older groups, with the LCA30 group demonstrating larger TBVs compared to the CON30 group. A two-way permutation ANOVA revealed a significant treatment × age interaction (*F*(1,27) = 5.84, *p* = 0.024), but no main effects [treatment: *F*(1,27) = 0.01, *p* = 0.938, age: *F*(1,27) = 2.77, *p* = 0.109)]. A bootstrapped *post hoc* analysis of significant main effects analyses revealed a difference between CON13 and CON30 (*p* = 0.020, 95% CI [7.87, 29.65]). See Supplementary Tables 10, 11 for descriptive statistics of TBVs and Supplementary Figure 12 for unnormalized ROI volumes.

**FIGURE 8:**
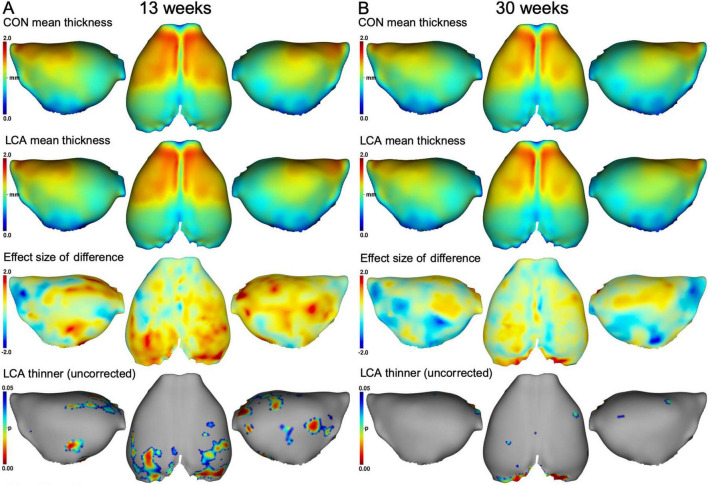
Mean cortex thickness maps in mm of panel **(A)** 13 weeks brains and **(B)** 30 weeks brains. First row: Group mean thickness of the CON groups. Second row: Group mean thickness of the LCA groups. Third row: Effect size of the differences between group means in the upper two rows (CON minus LCA). Fourth row: p-maps of where the LCA groups have thinner cortex, thresholded at 0.05 before correcting for multiple comparisons. No significant difference was observed after correction (see Supplementary Figure 14). See Supplementary Figure 13 for mean cortex thickness of each cortex ROI in all groups and Supplementary Table 12 for descriptive statistics.

We also investigated the cortex thickness of four cortical regions. Voxel-wise comparisons of the two groups suggested a difference in occipital cortex thickness at both ages (Figures 8A, B). However, after correcting for multiple comparisons, no significant differences were observed (Supplementary Figure 14). Comparisons of the ROIs revealed an age-related difference but also a treatment effect, mostly pronounced in the occipital cortex (Supplementary Figure 14), in agreement with the difference maps of Figure 8. Using three-way mixed permutation ANOVA to test the mean thickness of each ROI, we found a significant treatment × ROI interaction (*F*(3,81) = 4.01, *p* = 0.009) and main effects (treatment: *F*(1,27) = 4.57, *p* = 0.042, age: *F*(1,27) = 85.87, *p* < 0.000, ROI: *F*(3,81) = 1283.84, *p* < 0.000). The bootstrapped *post hoc* test of the treatment × ROI interaction found a significant difference between the two treatments in the occipital cortex (*p* < 0.000, 95% CI [0.05, 0.10]). Additionally, a bootstrapped *post hoc* test of the age effect revealed a significant difference in all ROIs (FC: *p* = 0.001, 95% CI [0.03, 0.08], PTC: *p* < 0.000, 95% CI [0.05, 0.07], OC: *p* < 0.000, 95% CI [0.05, 0.10], EC: *p* < 0.000, 95% CI [0.03, 0.07]). See Supplementary Figure 13. No significant interaction was observed for treatment × age × ROI (*F*(3,81) = 0.45, *p* = 0.721), age × ROI (F(3,81) = 1.02, p = 0.398), or treatment × age (*F*(1,27) = 1.36, *p* = 0.254). See Supplementary Table 12 for descriptive statistics.

#### 3.3.3 Mean kurtosis

The diffusion MRI-derived indices of tissue water mobility (MD), anisotropy (FA), and microstructural complexity (MK) were compared for all groups. See Supplementary Figures 15–18 for examples of DKI maps for a coronal slice in each metric and animal.

**FIGURE 9:**
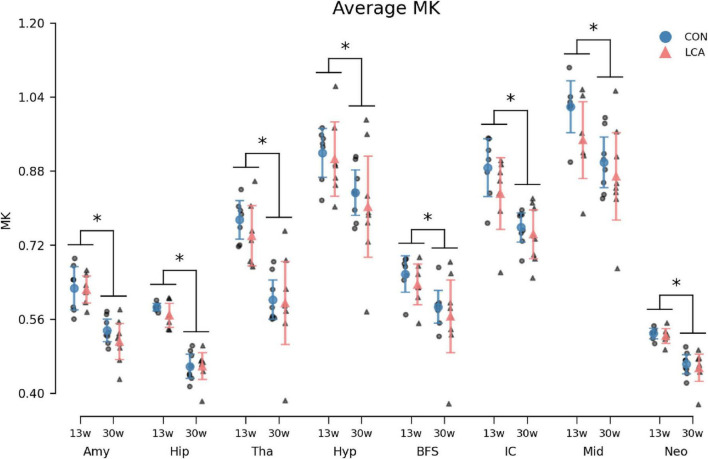
Average MK in the eight ROIs for each of the four groups. Group means with 95% CI are superimposed on the individual observations. In all ROIs, an age-related decrease in MK was observed, but no difference was seen between treatment groups. See Supplementary Table 13 for descriptive statistics. 13w, 13 weeks old; 30w, 30 weeks old; Amy, amygdala; hip, hippocampus; tha, thalamus; hyp, hypothalamus; BFS, basal forebrain septum; IC, inferior colliculi; neo, neocortex; mid, midbrain; tha, thalamus. *Age difference (*p* < 0.05).

In general, we observed a decline in the MK with age within all ROIs (Figure 9). While the LCA groups exhibited reduced MK compared to the CON groups, this downward shift was not significant. The three-way mixed permutation ANOVA unveiled a significant main effect of age (*F*(1,26) = 29.28, *p* < 0.000) and ROI (*F*(7,182) = 555.08, *p* < 0.000), as well as their interaction (*F*(7,182) = 4.25, *p* < 0.000), while the remaining terms (treatment: (*F*(1,26) = 1.42, *p* = 0.246), treatment × age: (*F*(1,26) = 0.09, *p* = 0.772), treatment ROI: (*F*(7,182) = 1.10, *p* = 0.362), and treatment × age × ROI (*F*(7,182) = 0.79, *p* = 0.602) yielded no significant findings. The *post hoc* test of significant age × ROI simple main effects analysis revealed age differences in amygdala (*p* < 0.000, 95% CI [0.074, 0.130]), hippocampus (*p* < 0.000, 95% CI [0.101, 0.138]), thalamus (*p* < 0.000, 95% CI [0.101, 0.138]), hypothalamus (*p* < 0.000, 95% CI [0.101, 0.138]), basal forebrain septum (*p* < 0.000, 95% CI [0.101, 0.138]), inferior colliculi (*p* < 0.000, 95% CI [0.060, 0.153]), neocortex (*p* < 0.000, 95% CI [0.052, 0.084]), midbrain (*p* < 0.000, 95% CI [0.038, 0.160]), thalamus (*p* < 0.000, 95% CI [0.112, 0.209]). See Supplementary Table 13 for descriptive group statistics.

#### 3.3.4 Mean diffusivity

**FIGURE 10:**
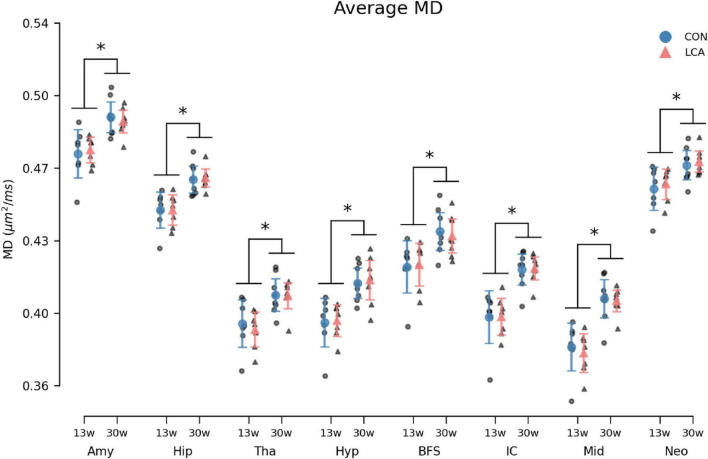
Average MD in the eight ROIs for each of the four groups. Group means with 95% CI are superimposed on the individual observations. Within all ROIs, the MD increases with age, but no differences are observed between the LCA and CON groups of the same age. See Supplementary Table 14 for detailed descriptive statistics. 13w, 13 weeks old; 30w, 30 weeks old; Amy, amygdala; hip, hippocampus; tha, thalamus; hyp, hypothalamus; BFS, basal forebrain septum; IC, inferior colliculi; neo, neocortex; mid, midbrain; tha, thalamus. *Age difference (*p* < 0.05).

MD increased by the age in all ROIs (Figure 10). However, no difference emerged between the LCA and CON groups. The outcomes of the three-way permutation ANOVA revealed the same significant terms as for the MK, encompassing age (*F*(1,26) = 28.23, *p* < 0.000), ROI (*F*(7,182) = 2354.00, *p* < 0.000), and their interaction (*F*(7,182) = 10.02, *p* < 0.008). No significant difference was observed for treatment (*F*(1,26) = 0.00), *p* = 0.97), treatment × age (*F*(7,182) = 0.00, *p* = 0.985), and the treatment × age × ROI (*F*(7,182) = 0.71, *p* = 0.669). The *post hoc* test of significant age × ROI simple main effects analysis revealed age differences in the amygdala (*p* < 0.000, 95% CI [−0.022, −0.010]), hippocampus (*p* < 0.000, 95% CI [−0.021, −0.011]), thalamus (*p* < 0.000, 95% CI [−0.023, −0.009]), hypothalamus (*p* < 0.000, 95% CI [−0.027, −0.013]), basal forebrain septum (*p* < 0.000, 95% CI [−0.024, −0.009]), inferior colliculi (*p* < 0.000, 95% CI [−0.031, −0.017]), neocortex (*p* < 0.000, 95% CI [−0.018, −0.006]), midbrain (*p* < 0.000, 95% CI [−0.032, −0.018]), and thalamus (*p* < 0.000, 95% CI [−0.023, −0.009]). See Supplementary Table 14 for descriptive group statistics.

#### 3.3.5 Fractional anisotropy

**FIGURE 11:**
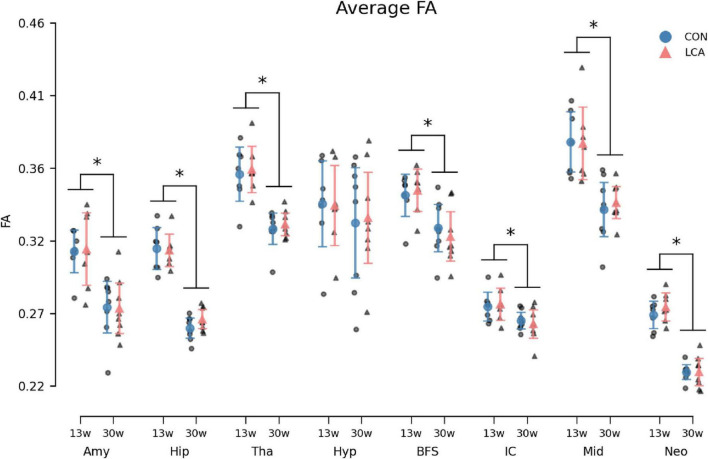
Average fractional anisotropy (FA) in the eight ROIs for each of the four groups. Group means with 95% CI are superimposed on the individual observations. Similar to the MK and MD, an age-related effect was observed across most ROIs. The LCA group did not deviate from the CON group of the same age. See Supplementary Table 15 for detailed descriptive statistics. 13w, 13 weeks old; 30w, 30 weeks old; Amy, amygdala; hip, hippocampus; tha, thalamus; hyp, hypothalamus; BFS, basal forebrain septum; IC, inferior colliculi; neo, neocortex; mid, midbrain; tha, thalamus. *Age difference (*p* < 0.05).

The FA showed concordant trends with the MK and the MD by exhibiting an age-related difference within the ROIs but with no difference between the treatments (Figure 11). The three-way permutation ANOVA found significant terms for age (*F*(1,26) = 46.35, *p* < 0.000), ROI (*F*(7,182) = 204.00, *p* < 0.000), and their interaction (*F*(7,182) = 6.59, *p* < 0.000). The treatment (*F*(1,26) = 0.07), = *p* = 0.793), treatment × age (*F*(7,182) = 0.00, *p* = 0.968), treatment × age × ROI (*F*(7,182) = 0.26, *p* = 0.971) revealed no significant effects. The *post hoc* test of significant age × ROI simple main effects analysis revealed age differences in amygdala (*p* < 0.000, 95% CI [0.025, 0.052]), hippocampus (*p* < 0.000, 95% CI [0.042, 0.057]), thalamus (*p* < 0.000, 95% CI [0.026, 0.047]), basal forebrain septum (*p* < 0.004, 95% CI [0.014, 0.037]), inferior colliculi (*p* < 0.004, 95% CI [0.005, 0.018]), neocortex (*p* < 0.000, 95% CI [0.034, 0.047]), midbrain (*p* < 0.000, 95% CI [0.028, 0.057]), thalamus (*p* < 0.000, 95% CI [0.026, 0.047]). See Supplementary Table 15 for descriptive group statistics.

## 4 Discussion

The LC is often attributed a role in trophic support of the brain, but its precise role in this is rarely specified. Our study compares brain volume and microstructure in control and LC-ablated mice without other pathology. This allows us to assess the effect of LC ablation on key components of trophic support, such as neuroplasticity, postnatal brain development, neuroprotection, and maintenance. LC ablation using DSP-4 is an established and robust method (Bortel, 2022; Ross and Stenfors, 2015), and its mechanisms are well-understood. Ablation via systemic (i.e., IP) administration of DSP-4 is possible because the compound easily crosses the blood-brain barrier, after which it cyclizes into the reactive aziridinium ion (Dudley et al., 1990). This ion is selectively taken up by NA nerve terminals, where it causes irreversible inhibition of NA transporters (Wenge and Bönisch, 2009), decreased ATP levels (Wenge and Bönisch, 2009), and degeneration of nerve terminals (Fritschy and Grzanna, 1989; Jonsson et al., 1981). Consequently, NA synthesis, release, transport, and metabolism are disrupted, leading to long-lasting LC neurodegeneration (Bortel, 2022; Ross and Stenfors, 2015). Compensatory mechanisms have been reported but do not reach pre-treatment levels until about a year after the last DSP-4 treatment (Fritschy and Grzanna, 1992; Puoliväli et al., 1999; Song et al., 2019; Wolfman et al., 1994). Repeated injections of DSP-4 suppress this mechanism (Heneka et al., 2010; Puoliväli et al., 1999), and DSP-4 treatment, as performed here, produces pronounced and consistent LC ablation (Markussen et al., 2023).

To further our understanding of LC’s role across developmental stages, we included two age groups. In the LCA13 group, ablation is induced as early as possible (at weaning), i.e., from 3 weeks of age. At this point, the noradrenergic system differs from the adult brain in terms of noradrenergic neurons, NA levels, and adrenergic receptor density (Murrin et al., 2007). The older cohort (LCA30) was ablated at 12 weeks of age, where the LC-NA system is fully developed (Murrin et al., 2007). Given the mechanisms of DSP-4 ablation described above, we do not expect to see similar effects of DSP-4 in these two age groups. Rather, we expect the LCA30 group to closely resemble the LCA groups investigated in Markussen et al. (2023), whereas the ablation effect in the LCA13 group is expected to be subtle because of less efficient DSP-4 uptake in the immature noradrenergic system, which does not reach adults levels until 5 weeks of age (Murrin et al., 2007). In these young mice, we also cannot rule out the capacity for more pronounced compensation. This should be taken into account when interpreting findings (see discussion of BM test results below). DKI analysis of the LC-containing region in pons is consistent with these considerations. In this region, microstructural alterations were found in LCA30 compared to CON30 (Figure 6D), with MK significantly decreased in LCA30. While unspecific, this decrease is likely due to cellular degeneration in the LC due to ablation. In the CON13/LCA13 groups, similar mean values of MK in the LC-containing region were observed but the distribution mode was lower in LCA13 than in CON13 and the distribution variances were significantly different (*p* = 0.007) (Figure 6B and supporting text). While the alignment between behavioral changes and MK measurements clearly confirms an ablation in the LCA30 group, the subtle behavioral alterations and diffuse impact on MK with increased MK variability in the LCA13 mice collectively suggest that DSP-4 effects in the immature noradrenergic system differ from the mature. The increased MK variability in LCA13 may indicate a tissue response of both cell death (causing decreased tissue complexity) and regional inflammatory response causing increased MK in some areas. One possible explanation for the different MK response in the LCA groups could be that in the older mice, the inflammatory/immune processes driving MK up in LCA13 have subsided with only MK decrease from cell death remaining. Different dynamics of DSP-4 have previously been observed by Jaim-Etcheverry and Zieher (1980), where neonatal treatment of DSP-4 induced long-lasting reduced NA levels in distant brain regions and increased levels in brain regions closer to LC. This indicates greater compensatory capacity in younger mice. We see the two groups as representing models of LC dysfunction during late-stage brain maturation (LCA13) and chronic LC dysfunction in the normal adult brain (LCA30).

To assess global ablation effect on brain structure and microstructure we used *ex vivo* MRI to perform whole-brain analysis. This was done using in-skull samples so that atlas-based segmentation can be performed with high fidelity. We use MR microscopy data for brain volumetric analysis and DKI for assessment of brain microstructure. Both methods are known to be sensitive to brain structure alterations as a response to neurotoxin-induced disease and drug use (Garza-Villarreal et al., 2017; Hansen, 2020) as well as subtle alterations in response to learning (Vukovic et al., 2021) or stress (Khan et al., 2016, 2018; Vestergaard-Poulsen et al., 2011).

### 4.1 LC ablation induces subtle behavioral changes

A comprehensive behavioral test battery was performed in the CON30 and LCA30 groups. The main purpose of these tests was *in vivo* confirmation of ablation effects by comparison with our previously reported behavior readouts from animals with histologically confirmed ablation (Markussen et al., 2023). Our test battery included the LDB test for assessing anxiety-like behavior (Kulesskaya and Voikar, 2014), and BM to test spatial learning and memory (Gawel et al., 2019). This was done at three different time points. Based on a preview of these results, we adjusted the test format in the CON13/LCA13 groups to only include a single time point. Figures 2–5 and Supplementary Figures 5–10 provide a graphical overview of these results.

In the LDB test of the older mice with the longest post-ablation period, the LCA30 group spent more time in the light compartment. This effect was most pronounced in the test carried out 1 week after treatment. Even though the *post hoc* test revealed no significant differences between the LCA30 and CON30 groups, these results are consistent with our previous findings (Markussen et al., 2023). Along with a slightly increased number of transitions between the compartments, the results of all LDB test metrics of the older cohort of mice generally mimic our previous findings. The effect of increased time in the light compartment was also present in the LCA13 group, but only during the last 5 min. The time spent in the light compartment reflects the innate aversion of mice to open, illuminated areas (Gawel et al., 2019; Kulesskaya and Voikar, 2014). However, as the innate drive to explore new environments is another important feature of the LDB test, this potentially confounds the effect of the LC ablation. Studies have shown that while NA-depleted mice indeed exhibit reduced anxiety-like behavior, these mice also lack typical novelty-induced behavior, such as increased locomotion (Lustberg et al., 2020). One might, therefore, expect these two opposite effects to cancel out to a degree where no effect of LC ablation would be observable in the LDB test. However, a group difference is observed with regard to time spent in the light compartment, indicating that reduced anxiety is more pronounced in our model than lack of novelty seeking. Nevertheless, the motor-related metrics showed less and slower movement in the light compartment 1 week after treatment (Supplementary Figure 6), which along with the increased time in the light compartment indicates a reduction of typical novelty-induced behavior.

During the BM test, the LCA30 and CON30 groups performed similarly on the third TD (Figure 4). This level of performance was retained in the 1-week post-treatment test, resulting in no notable differences between the groups. However, the 8-week post-treatment test showed an increased number of errors in the LCA30 group compared to the CON30 group (Figure 4I). The learning curve was comparable to the pre-treatment performance, while the performance of the CON30 group 8 weeks after treatment was similar to their performance 1 week after treatment. Additionally, looking at the search strategy metrics, the LCA30 group visits an increased number of different non-target zones compared to the CON30 group (Figure 4C), indicating a more arbitrary search strategy (Illouz et al., 2016), while also spending more time in the quadrant of the BM table opposite the escape box (Figure 5I). This, too, agrees with our previous findings (Markussen et al., 2023). While not statistically significant, we observed an increase in the number of errors and distance covered in the LCA30 group during the PD 8 weeks after the treatment (Supplementary Figures 8A–D). LC ablation has been found to affect learning mainly in novel contexts (Takeuchi et al., 2016; Wagatsuma et al., 2018). However, the longer period between the first and second post-treatment tests may be sufficiently long for task retention to be lost. No differences were observed between the LCA13 and CON13 groups in the BM test during the TDs nor PD (Supplementary Figures 9, 10). Iannitelli et al. (2023) have previously reported behavioral outcomes of DSP-4 treatment indicating the presence of mechanisms acting to compensate for the effect of the LC ablation. We believe that this may reflect a more pronounced compensation as in mice with early LC ablation compared to mice with later LC ablation as outlined above. Overall, the behavioral findings of the LCA30 group are consistent with behavior observed in mice with histologically confirmed LC ablation (Markussen et al., 2023), while the effects of DSP-4 are less pronounced in the LCA13 group.

### 4.2 LC ablation causes no brain volume alterations

Our volumetric analysis indicated no significant differences in regional brain volumes between the LCA and CON groups. This was the case for early LC ablation at 3 weeks of age and for older mice at 12 weeks of age. Comparing our volumetric results with previous studies can be challenging as these often report absolute rather than relative sizes of brain region volumes (Badea et al., 2007, 2009; Dorr et al., 2008; Hammelrath et al., 2016; Ma et al., 2005, 2008; Totenhagen et al., 2017). However, because the size of the brain regions strongly correlates with measures of brain volume (Badea et al., 2009; Lerch et al., 2012), adjusting for brain size is recommended (O’Brien et al., 2011). This is both preferable when comparing regional volumes across studies as TBV estimates vary substantially (Aggarwal et al., 2009; Badea et al., 2009; Hammelrath et al., 2016; Holmes et al., 2017; Kooy et al., 1999; Kovaèeviæ et al., 2005; Ma et al., 2005, 2008; Maheswaran et al., 2009; Qvist et al., 2018; Totenhagen et al., 2017; Zhang et al., 2010), but also for effectively reducing the within-group variability caused by natural biological variation and, thus, increasing the statistical power (Lerch et al., 2012).

The TBV variability between studies is attributable to multiple factors, including segmentation method, genetic strain differences (Badea et al., 2009), and brain tissue preparation. Longitudinal studies have reported 3–10% reduction in TBV between *in vivo* to *ex vivo* (Aggarwal et al., 2009; Holmes et al., 2017; Zhang et al., 2010). While fixation typically causes brain tissue to shrink (Gros et al., 2023), these alterations are not uniform among brain regions (Holmes et al., 2017; Ma et al., 2019; Ma et al., 2008; Zhang et al., 2010), making simple scaling of brain regions unsuitable for direct comparisons of *in vivo* and *ex vivo* imaging. Ex vivo, in-skull imaging, however, appears to have a preserving effect on central brain structures, limiting deformations to mainly affect the olfactory bulb and brainstem (Holmes et al., 2017).

Interestingly, despite having reached an age where brain volume is typically seen to plateau (Aggarwal et al., 2009; Hammelrath et al., 2016; Zhang et al., 2010), we observed a difference in TBV between the two CON groups (Supplementary Figure 11A). However, as these findings align with the expected variability reported in extensive studies (Wang et al., 2020), we consider this TBV difference insignificant. After adjusting the brain regions to TBV, no regional differences between the groups were observed. Additionally, although the TBV estimates reported by Maheswaran et al. (2009) are on the high end of the literature, the relative volumes of the hippocampus, thalamus, and hypothalamus are still similar to ours. This reaffirms the strong correlation between TBV and regional volume. As an example, Supplementary Figure 11B shows the correlation between TBV and hippocampus volume seen in our data.

Few studies have explored the effects of LC ablation on brain structures other than the LC. Maheswaran et al. (2009) found no impact of the LC ablation on brain regional volume in either wild-types or Alzheimer’s disease models. Notably, the neurodegenerative disease model exhibited larger TBV and regional volumes compared to the control group, contradicting the usual findings of neurodegenerative models exhibiting considerable volumetric reductions (Holmes et al., 2017; Ma et al., 2019; Zhang et al., 2010), and, therefore, warrants further follow-up studies. Additionally, Heneka et al. (2006) observed increased pathogenesis in transgenic Alzheimer’s mice treated with DSP-4 pointing to LC dysfunction promoting neurodegeneration in the presence of disease.

Ventricular volume expansion typically indicates neurodegeneration (Ma et al., 2019; Nestor et al., 2008; Weiner, 2008; Zhang et al., 2010) and could, therefore, be a potential biomarker. However, ventricles are highly susceptible to deformation after perfusion fixation as cerebrospinal fluid displacement causes them to collapse (Ma et al., 2008; Zhang et al., 2010). Therefore, we have not included the ventricles as a ROI in our volumetric analysis. Furthermore, one could expect the TBV shrinkage from *in vivo* to *ex vivo* imaging to be greater in the LCA groups and potentially affect the ROI volume estimates relative to the TBV. However, the TBV shrinkage following tissue fixation appears homogeneous when comparing wild-types and tauopathy models (Holmes et al., 2017). I.e., the TBV reduction following perfusion fixation in disease models with expanded ventricles is similar to wild types. We therefore do not find reason to believe that such effects impact our results.

### 4.3 Microstructural changes from LC ablation not detectable by DKI

After our volumetric analysis, we assessed brain microstructure using DKI. This method is a clinically applicable diffusion MRI method sensitive to brain cytoarchitecture and is increasingly used in the study of neurodegenerative diseases (Spotorno et al., 2024). However, no studies have investigated the impact of LC ablation on brain microstructure with DKI. Age-related effects on DKI parameters are often studied in longitudinal *in vivo* studies. Our study is performed *ex vivo* which makes comparison difficult because of the differences in diffusion properties between brain tissue *in vivo* and in the fixed state (Schilling et al., 2022). When appropriate we will, however, discuss *in vivo* findings and how they might relate to our findings.

Our MD estimates showed no differences between the LCA and CON groups. However, a main effect of age was observed. The MD increased across all ROIs from 13 to 30 weeks in line with existing literature (Cheung et al., 2009; Nie et al., 2015; Taylor et al., 2020). Our MD estimates are consistent with other *ex vivo* studies (Khan et al., 2018). Note, however, that MD is typically higher *in vivo*, meaning that caution is needed when comparing *in vivo* findings to *ex vivo* (Falangola et al., 2022; Khairnar et al., 2017; Zerbi et al., 2013). As LC pathology often precedes neurodegeneration (Braak and Del Tredici, 2011; Vermeiren and De Deyn, 2017), one could speculate that early stages of Alzheimer’s and Parkinson’s disease are comparable with LC ablation-induced pathology. Manno et al. (2019) were capable of detecting microstructural alterations in the cortex of an Alzheimer’s disease mouse model at 2 months of age, and Falangola et al. (2020) found MD differences in a similar disease model and age group. However, such microstructural alterations are not visible in our results.

The FA was not different between the LCA and CON groups, but as for the MD, age also affects the FA. In agreement with previous studies (Cheung et al., 2009; Hammelrath et al., 2016; Taylor et al., 2020), our estimates of FA generally decrease with age in all investigated ROIs. It should be noted that the previous studies report an initial increase in FA during the first postnatal weeks, but this time point is not included in our study. It is not entirely surprising to find that FA is unaffected by LC ablation, as previous studies of FA alterations in mouse models of neurodegenerative diseases show contradictory effects or an ambiguously affected FA. In a model of tauopathy, FA did not differ from controls in the hippocampus, thalamus, and amygdala up to 8 months of Khairnar et al. (2017), Sahara et al. (2014). Contrary to this, studies have also reported increased FA in the hippocampus of young (Manno et al., 2019) and the cortex of old (Falangola et al., 2022) Alzheimer’s mice models.

We observed no difference in MK between the LCA and CON groups of the same age group except in the LC-containing part of pons (Figure 6). However, the data showed a statistically significant decrease in MK from 13 to 30 weeks in all ROIs. This finding contrasts previous *in vivo* rodent studies, where MK estimates typically develop with an inverted U-shape with age (Cheung et al., 2009; Han et al., 2021; Nie et al., 2015; Praet et al., 2018), and in humans, where MK has been reported to increase with age in the healthy brain (Garza-Villarreal et al., 2017). However, as for the MD and FA estimates, fixation-induced changes should be considered when comparing *in vivo* to *ex vivo* imaging results. Our MK estimates agree with previous *ex vivo* DKI estimates in control mice (Khan et al., 2018), and we, therefore, interpret these as biologically credible. Generally, comparisons of DKI and volumetric results between studies should account for the effects of measurement and analysis schemes (Chuhutin et al., 2020) and fixation effects, including protein cross-linking (Tayri-Wilk et al., 2020), lower experimental temperature (Thelwall et al., 2006), altered relaxation properties, increased membrane permeability (Shepherd et al., 2009), and general water displacement (Korogod et al., 2015). These effects cause differences between *in vivo* and *ex vivo* in measurements of water mobility (DKI) and regional volumes. However, as our comparisons are all performed under identical *ex vivo* conditions, these considerations are only relevant when comparing our findings to other studies.

Our results showed an ablation-related decrease in occipital cortex thickness as well as a general age-related decrease (Figure 8 and Supplementary Figure 13). It has previously been found that DSP-4 especially affects the NA concentration in the occipital cortex (Hallman et al., 1984). LC ablation would be expected to cause a decrease in cortical thickness as LC dysfunction would cause a loss of cortical maintenance from LC. In humans, lower LC integrity has been associated with lower cortical thickness (Engels-Domínguez et al., 2024), but it is unclear if there is a causative association or if one or more underlying processes drive both effects. The LC cell count is often said to decline with age (German et al., 1988; Manaye et al., 1995). However, it remains unclear whether an age-related decline in LC cell count exists when samples with existing pathology are excluded from the analysis (Kubis et al., 2000; Mouton et al., 1994). Importantly, studies using unbiased estimation procedures (Mouton et al., 1994; Ohm et al., 1997; Theofilas et al., 2017) did not find age-related differences, suggesting that the healthy brain retains the LC during aging (see Knopper and Hansen (2023) for review). However, these differences should be seen in the light of our raw data resolution (50 μm isotropic), meaning that the detection of differences less than the voxel size should be interpreted with caution even at group level. Nevertheless, our findings may serve as a valuable reference for future studies. The visual cortex is known to undergo final maturation at postnatal day 35 in mice (Katz and Shatz, 1996). It is, therefore, biologically plausible that the LCA13 groups would display cortical thickness differences in this region if LC were needed for such maturation. When inspecting Figure 8, indeed, the LCA13 group seems more affected than the LCA30 group. However, the visual cortex is known to preserve its plastic capacity to a greater extent than other brain regions (Knopper and Hansen, 2023), for which reason the weaker effect seen in the LCA30 group also seems plausible.

The absence of trophic support from the LC could potentially lead to microstructural alterations in the brain for several reasons. Exacerbated inflammatory responses (Heneka et al., 2010), reduced plasticity (Glennon et al., 2023), and diminished neuroprotection (Liu et al., 2015) due to NA suppression could disrupt the structural integrity of the brain and potentially decrease diffusional heterogeneity. NA is also known to suppress the glymphatic clearance (Hussain et al., 2023; Xie et al., 2013), which could affect the build-up of waste products in an imbalanced glymphatic system. The resulting tissue response to waste build-up would, in turn, affect water diffusion and DKI metrics. For instance, Khairnar et al. (2017) used DKI to detect protein aggregation in the thalamus of overexpressing α-synuclein mice. Furthermore, LC integrity has been found to reflect gray matter microstructural alteration (Elman et al., 2022) and neurodegenerative pathology progression (Bueichekú et al., 2024; Galgani et al., 2024). As the LC constitutes an early site for neurodegenerative disease pathology, such findings might suggest LC pathology to be a driving force in the development of neurodegenerative diseases. However, our results instead suggest that LC dysfunction in isolation does not produce brain microstructure alterations. Our results, therefore, suggest caution when interpreting an observed correlation between cortical alterations and LC integrity (indirectly assessed by neuromelanin MRI signal intensity). Specifically, such correlation should not immediately be seen as a causal relationship where loss of LC integrity causes gray matter changes. Rather, our findings suggest that it is more likely that the same underlying driving force produces both loss of LC integrity and gray matter alterations. This would explain why we do not observe similar effects in our study of LC ablation without comorbidity.

Studies on LC ablation in healthy rodents have previously shown contradictory results. While LC ablation indeed exacerbates β-amyloid plaque aggregation in Alzheimer’s mouse models (Kalinin et al., 2007), studies also suggest that LC ablation in wild-types does not invoke inflammatory responses (Heneka et al., 2006). This indicates that comorbidity may be required for LC ablation to negatively impact brain physiology, mainly because compensatory mechanisms sufficiently meet the NA demand of a brain with no simultaneous morbidity. These mechanisms encompass increased NA synthesis by upregulating tyrosine hydroxylase in the remaining LC neurons (Szot et al., 2000), increased post-synaptic adrenergic receptor density (Mogilnicka, 1986; Szot et al., 2007), and sprouting of surviving LC neurons (Fritschy and Grzanna, 1992). However, Song et al. (2019) found reduced neuronal count in the frontal cortex, hippocampus, and midbrain in wild-types treated with DSP-4. Our findings do not contradict these previous studies, indicating that inflammatory responses are affected by the LC, especially in disease models.

Our study uses only male mice to avoid the effects of the estrous cycle. This might shadow some effects as estrogens are known to have neuroprotective effects (Behl, 2002; Wise, 2002) and specifically interact with LC neurons (Heritage et al., 1977; Sar and Stumpf, 1981). Future studies should include females to also shed light on this important aspect, especially in studies where LC ablation is combined with pathology such as Alzheimer’s disease.

Our study employs whole-brain methods previously proven capable of detecting subtle morphological changes (Qvist et al., 2018) and brain microstructural remodeling in stress (Khan et al., 2018), learning (Vukovic et al., 2021), and disease (Falangola et al., 2022; Khairnar et al., 2016). We, therefore, believe that the MRI methods employed here are representative of the current detection threshold of MRI for whole-brain volumetric (including cortical thickness) and microstructural alterations. Despite the sensitivity of the MRI methods, we detect only a few ablation-related differences between groups. Nevertheless, the ablation will affect the brain-wide LC-NA network (Chandler et al., 2019; Liebe et al., 2022) as evidenced by its impact on behavior, discussed above and in Markussen et al. (2023). Earlier studies have shown histological changes in LC-ablated animals. These changes include NA fiber degeneration, oxidative stress (3-NT), astrocyte and microglial immunoreactivity (GFAP, Iba-1) (Iannitelli et al., 2023), but other studies have found no effects on microglia and astrocytes with subcellular effects being pronounced (Duffy et al., 2019). It seems, therefore, that the ablation effects, which cause behavioral changes, produce tissue alterations too subtle for detection with our MRI methods. Similarly, *in vivo* optical imaging in LC-ablated mice would likely show altered pericyte function (Korte et al., 2023) with potential consequences for tissue oxygen supply (Østergaard, 2020). Based on this, one might speculate that “omics” analyses would reveal alterations in cellular function and that longer-term LC ablation could manifest as tissue alterations detectable by MRI or MR spectroscopy. As it stands, however, our study shows that brain maintenance in the normal adult mouse brain (LCA30) occurs normally, even in the absence of LC-NA. Interestingly, our young mice (LCA13) show decreased thickness of the occipital cortex. This would indicate that brain maturation and growth are affected by LC ablation. As seen from our LCA30 group, behavior is affected, but regional brain volumes and microstructure are unaffected by LC ablation. Our study adds clarity to the often-encountered notion that LC-NA is important for brain trophic support as it shows that such effects are most likely more relevant to mechanisms related to brain development than for maintenance of the normal, young brain. The literature indicates that LC is important for disease response (neuroprotection) and brain plasticity with a strong driver. If this is true, then our study, which does not include a disease process or a driver for plasticity [e.g., learning task (Vukovic et al., 2021) or stress (Khan et al., 2016, 2018; Vestergaard-Poulsen et al., 2011)], would not be expected to show effects of LC ablation in the older cohort. The aging (but otherwise healthy) brain may be less robust to LC dysfunction than the brains of the young adult mice used here. This should be explored in later work. Future studies should also expand this work to contrast the effects of isolated LC ablation with the impact of LC ablation in combination with other pathologies such as trauma (Hussain et al., 2023), stroke, and Alzheimer’s disease.

Through LC ablation in the otherwise healthy brain, this study provides insight into LC dysfunction in isolation, which cannot be studied in patients. Our study shows that LC-NA is not vital for postnatal brain maturation, growth, or brain maintenance in the healthy adult mouse brain. Instead, LC-NA is likely more critical in the “challenged” brain for mechanisms related to brain plasticity or neuroprotection in response to disease.

## Data Availability

The raw data supporting the conclusions of this article will be made available by the authors, without undue reservation.
