# Supplementary material for: The effects of locus coeruleus ablation on mouse brain volume and microstructure evaluated by high-field MRI
Source: Front Cell Neurosci. 2024 Dec 11;18:1498133. doi: 10.3389/fncel.2024.1498133 (PMC11668759; doi:10.3389/fncel.2024.1498133)
Supplement: Supplementary file 1 [file Data_Sheet_1.pdf]

Supplementary Material

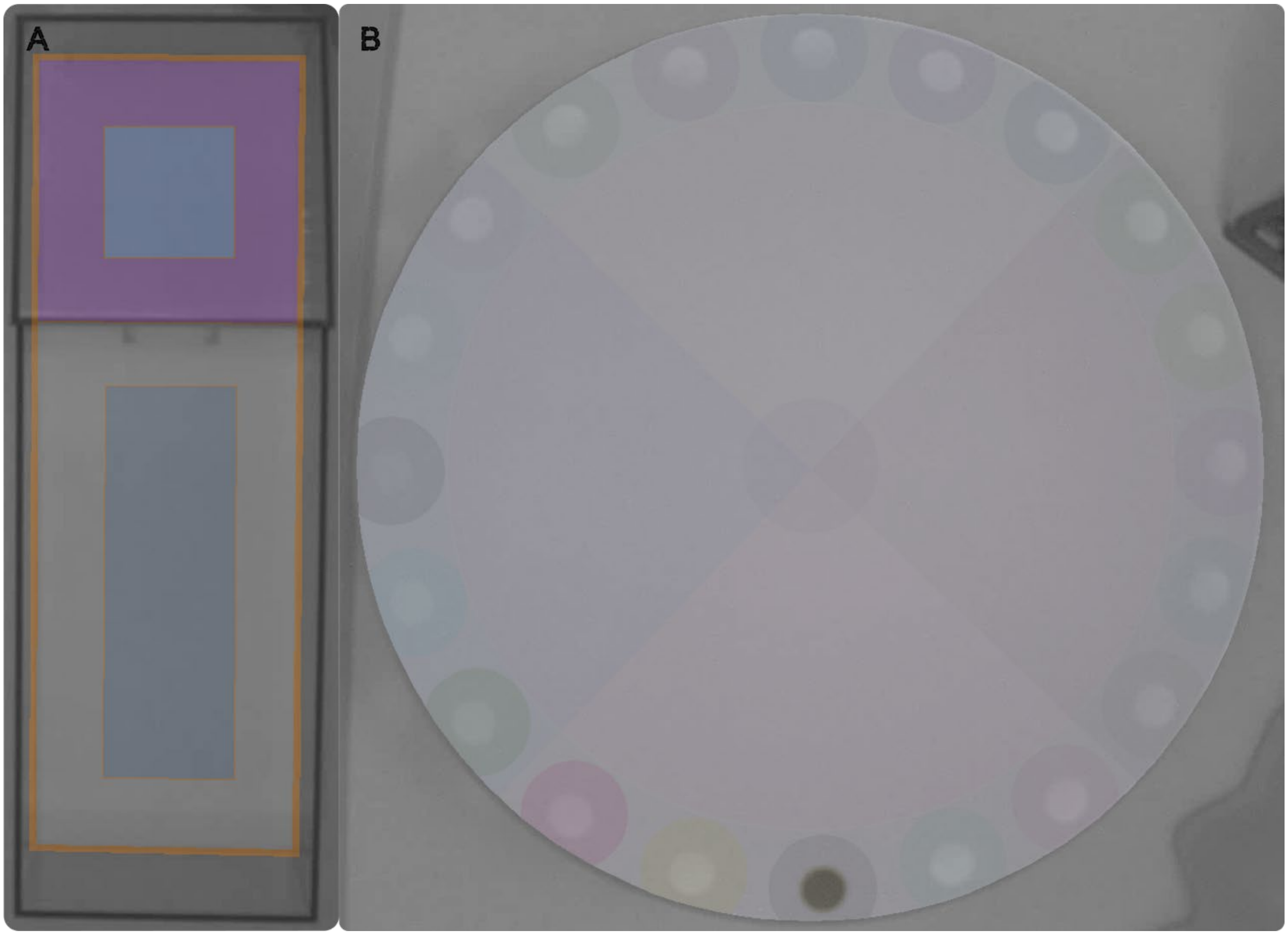

Supplementary Figure 1: Examples of how the areas of the behavioural tests were defined. **(A)** The light-dark box (LDB) test arena. The small compartment was the dark area, and the large compartment was the light area. An inner and outer area were defined in both compartments. **(B)** The Barnes maze (BM) test arena. The circular table had multiple exit holes but only one of them had an escape box underneath (appears black in the image). The table was divided into four quadrants with entrance zones around each hole.

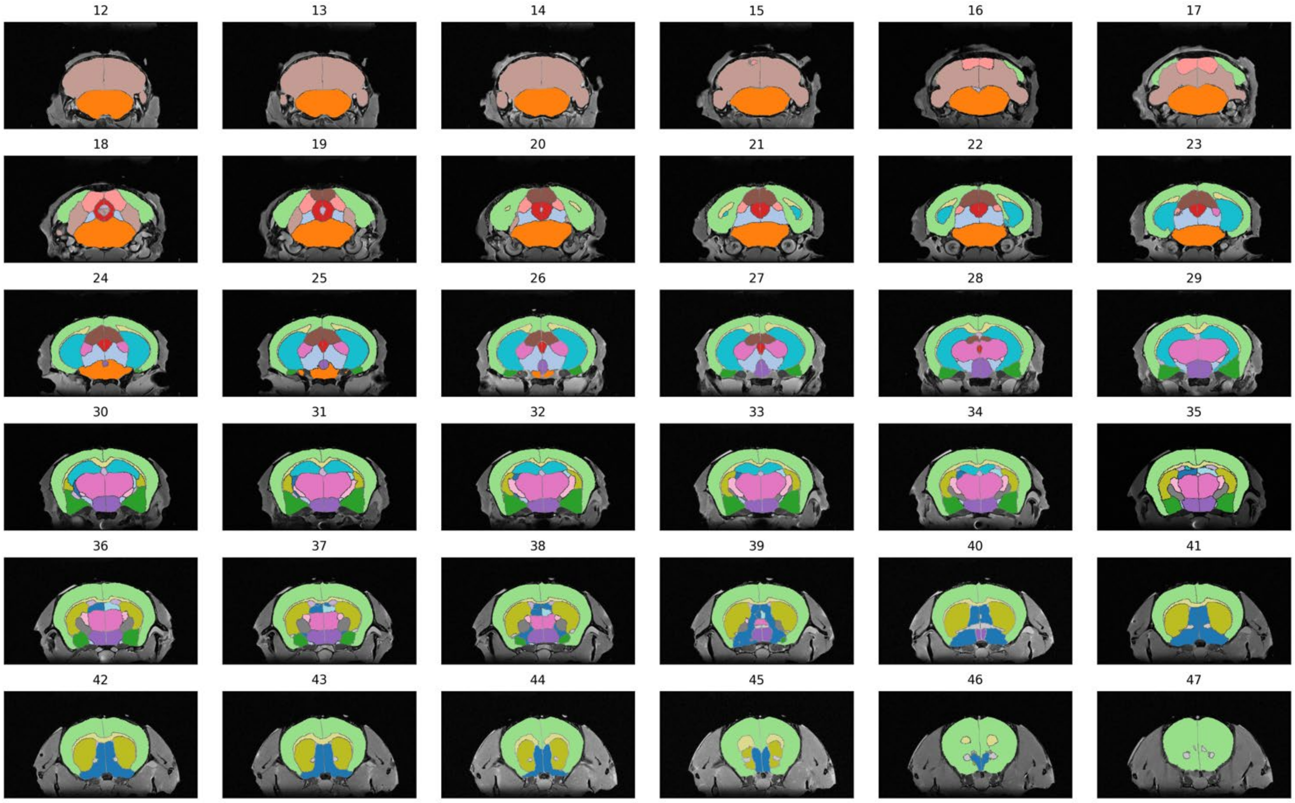

Supplementary Figure 2: Example of a segmented brain using MAS.

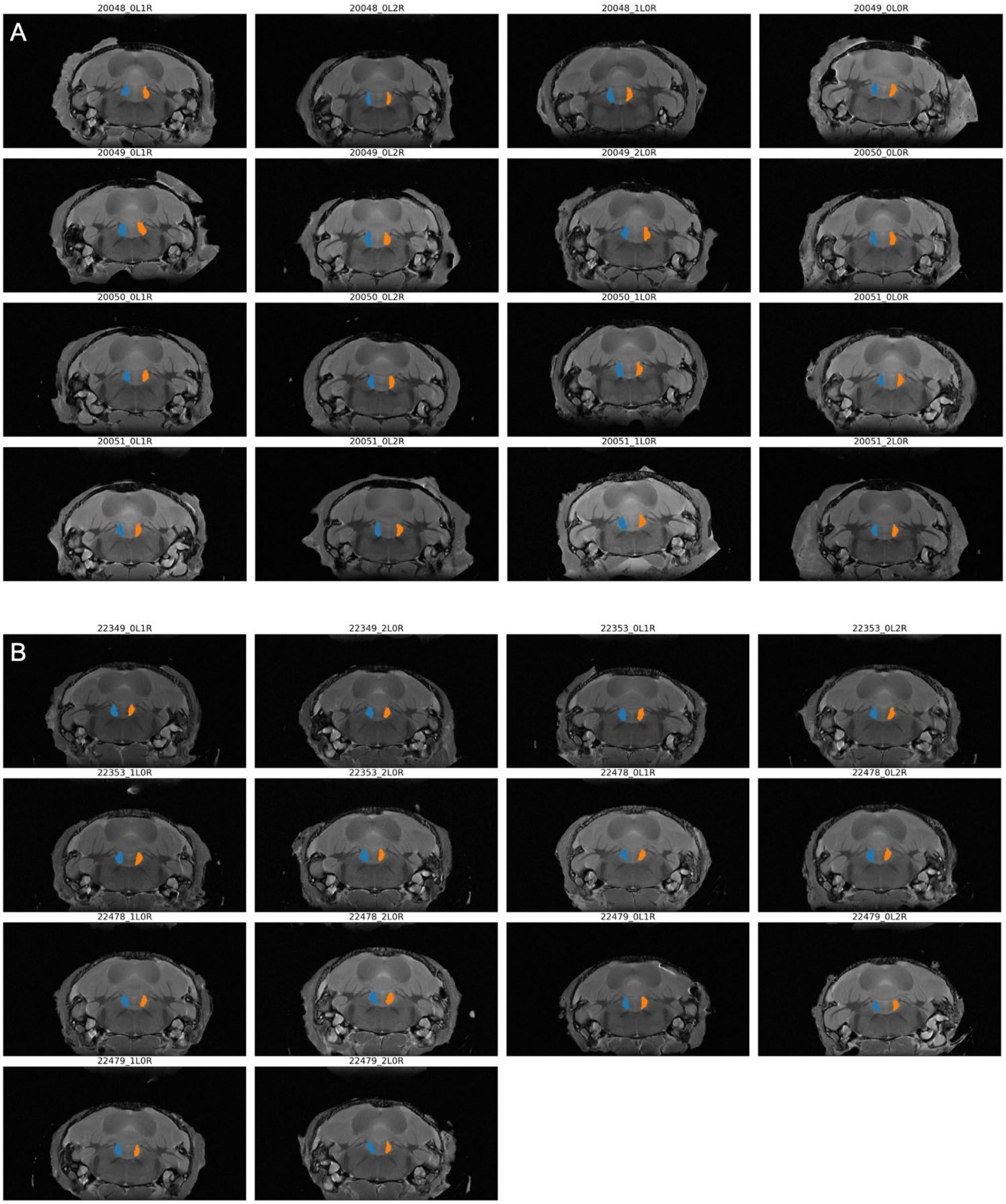

Supplementary Figure 3: LC-containing ROIs for each animal. (A) The mice of the CON30 and LCA30 groups. (B) The mic of the CON13 and LCA13 mice.

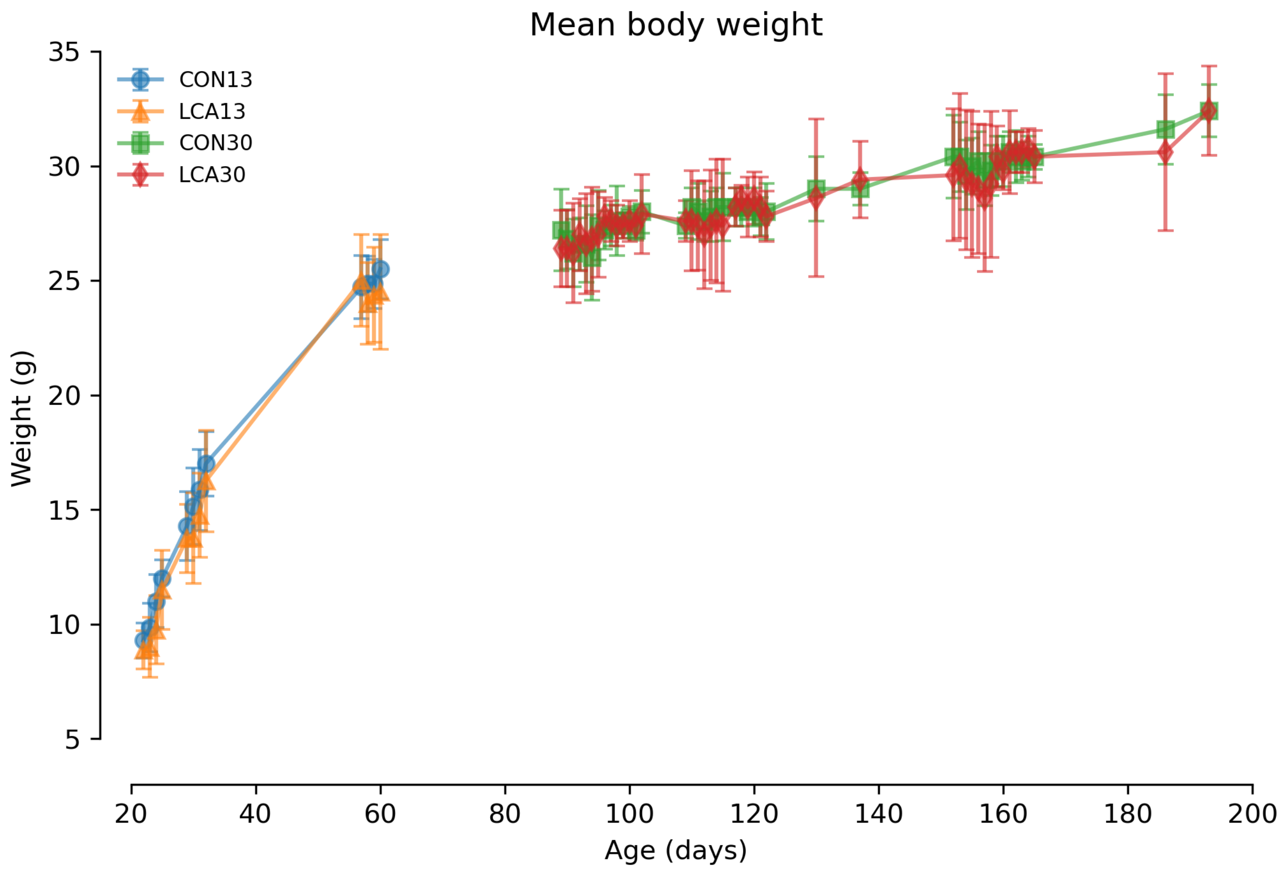

Supplementary Figure 4: The group mean body weight of the four groups. The readings of body weight were related to treatment and/or behavioral testing. Error bars = SD.

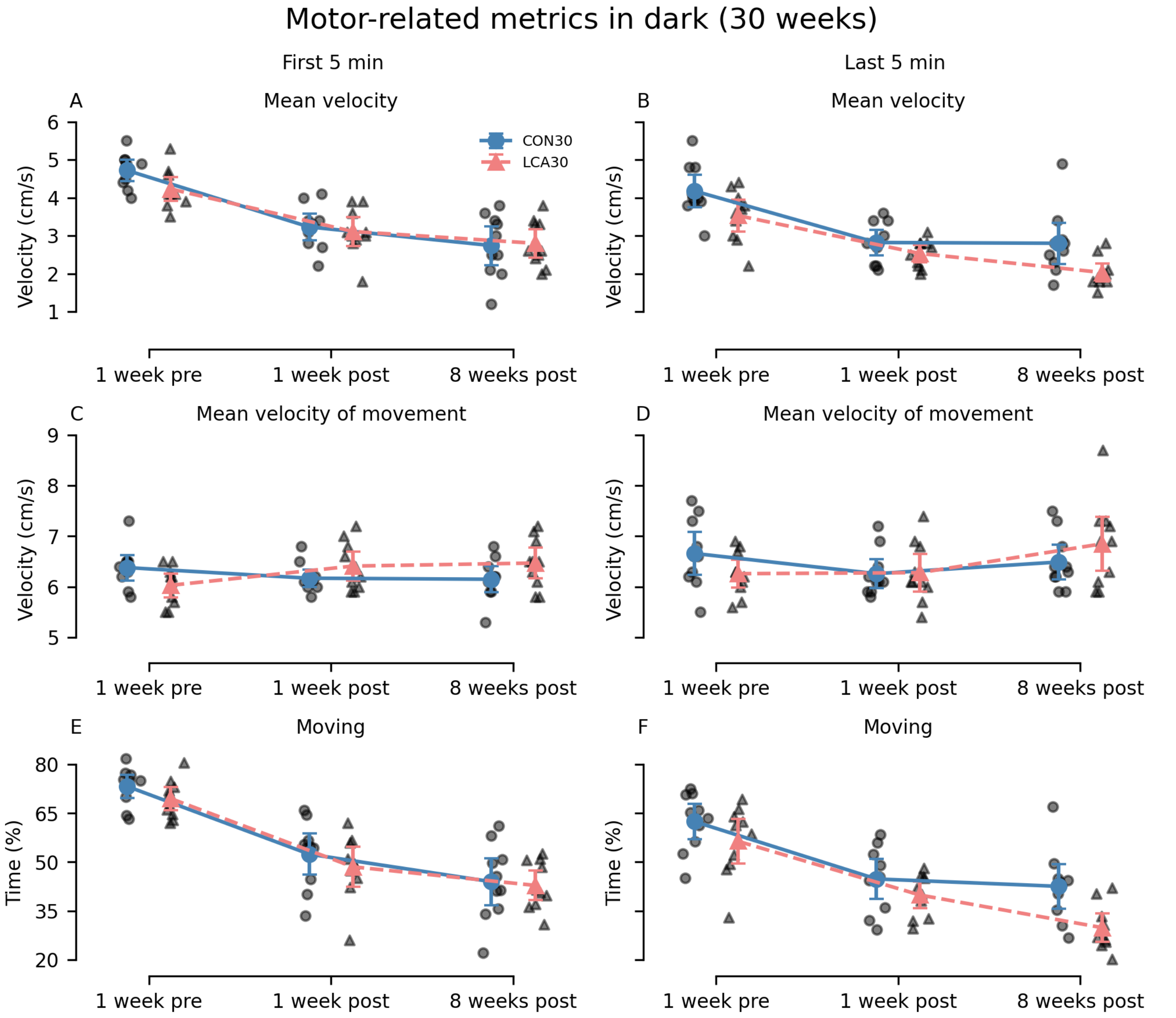

Supplementary Figure 5: The three motility parameters measured in the dark compartment and divided into time bins of five minutes. The x-axis shows the time relative to the time of treatment.

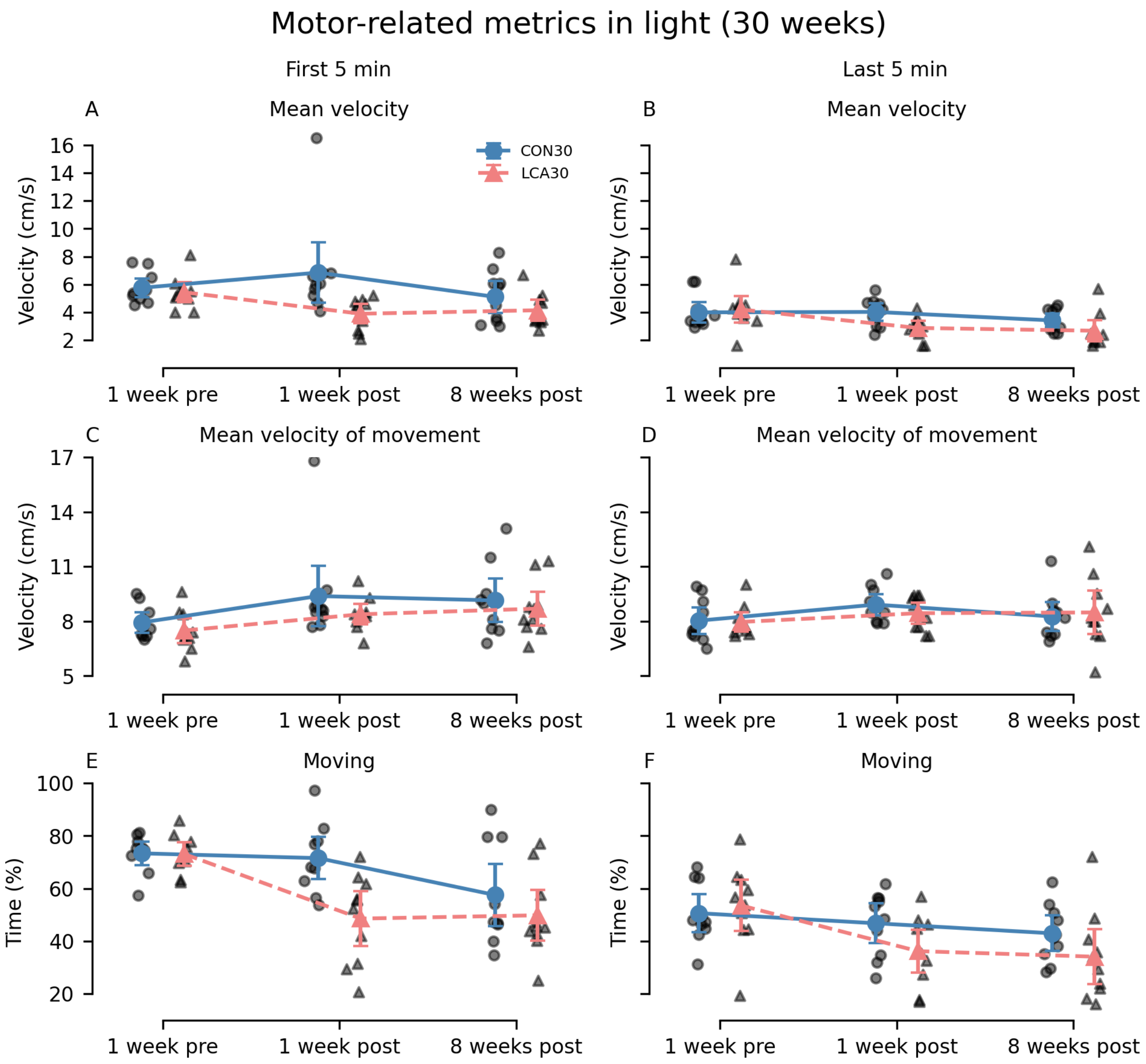

Supplementary Figure 6: The three motor-related metrics of the CON30/LCA30 groups measured in the light compartment and divided into time bins of five minutes. The x-axis shows the time relative to the time of treatment.

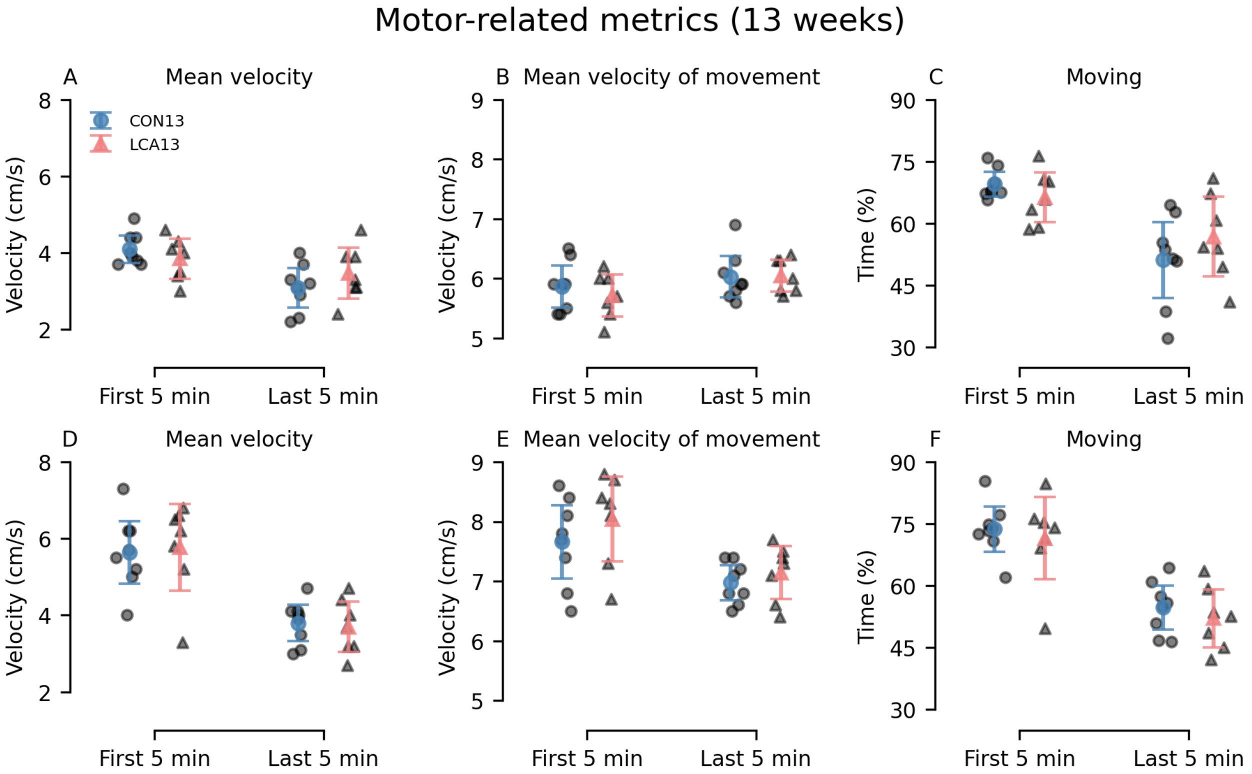

Supplementary Figure 7: The three motor-related metrics of the CON13/LCA13 groups measured in the dark compartment (A-C) and in the light compartment (D-F) divided into time bins of five minutes. The x-axis shows the time relative to the time of treatment.

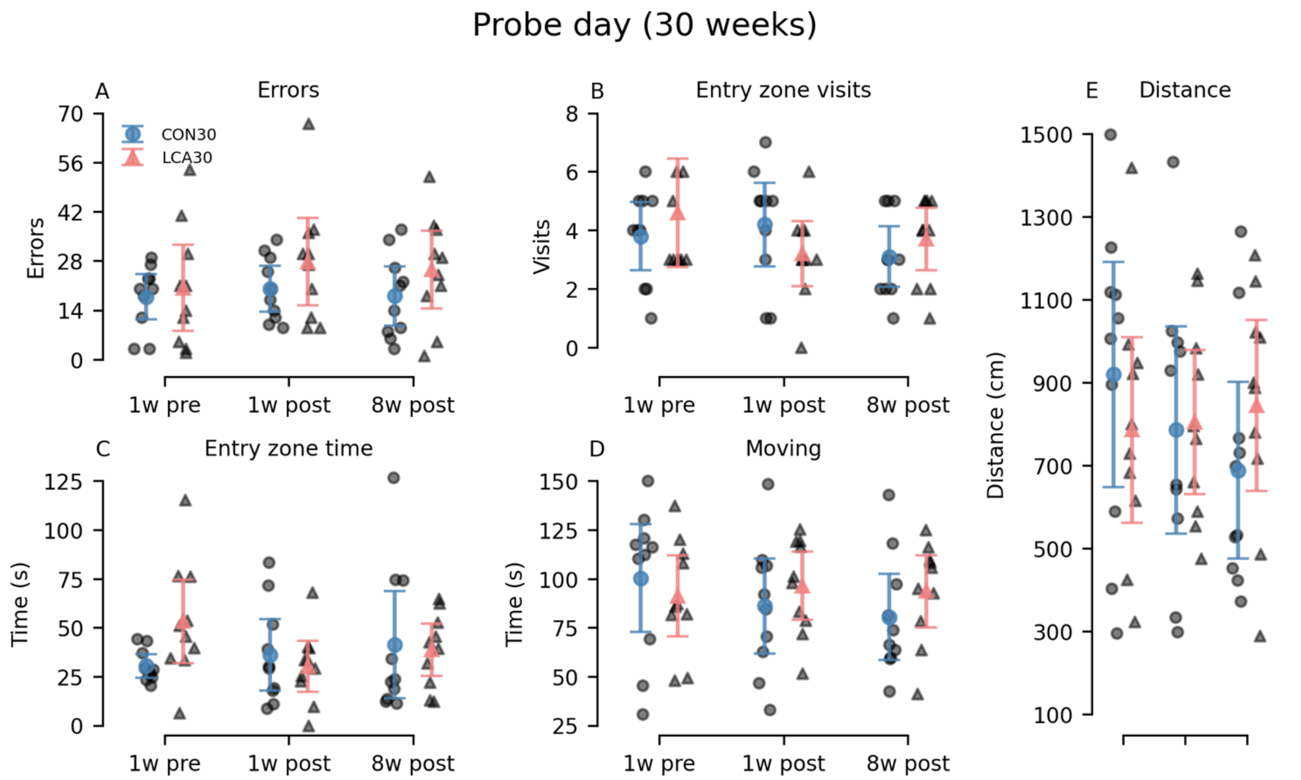

Supplementary Figure 8: The Barnes maze (BM) results of the 30-week-old mice on the probe days (PDs) at each of the three time points. The x-axis shows the time relative to the time of treatment. Note, that the x-axis of (E) is identical to the others. No differences were observed between the two groups. 1w pre = one week before treatment, 1w post = one week after treatment, 8w post = eight weeks after treatment.

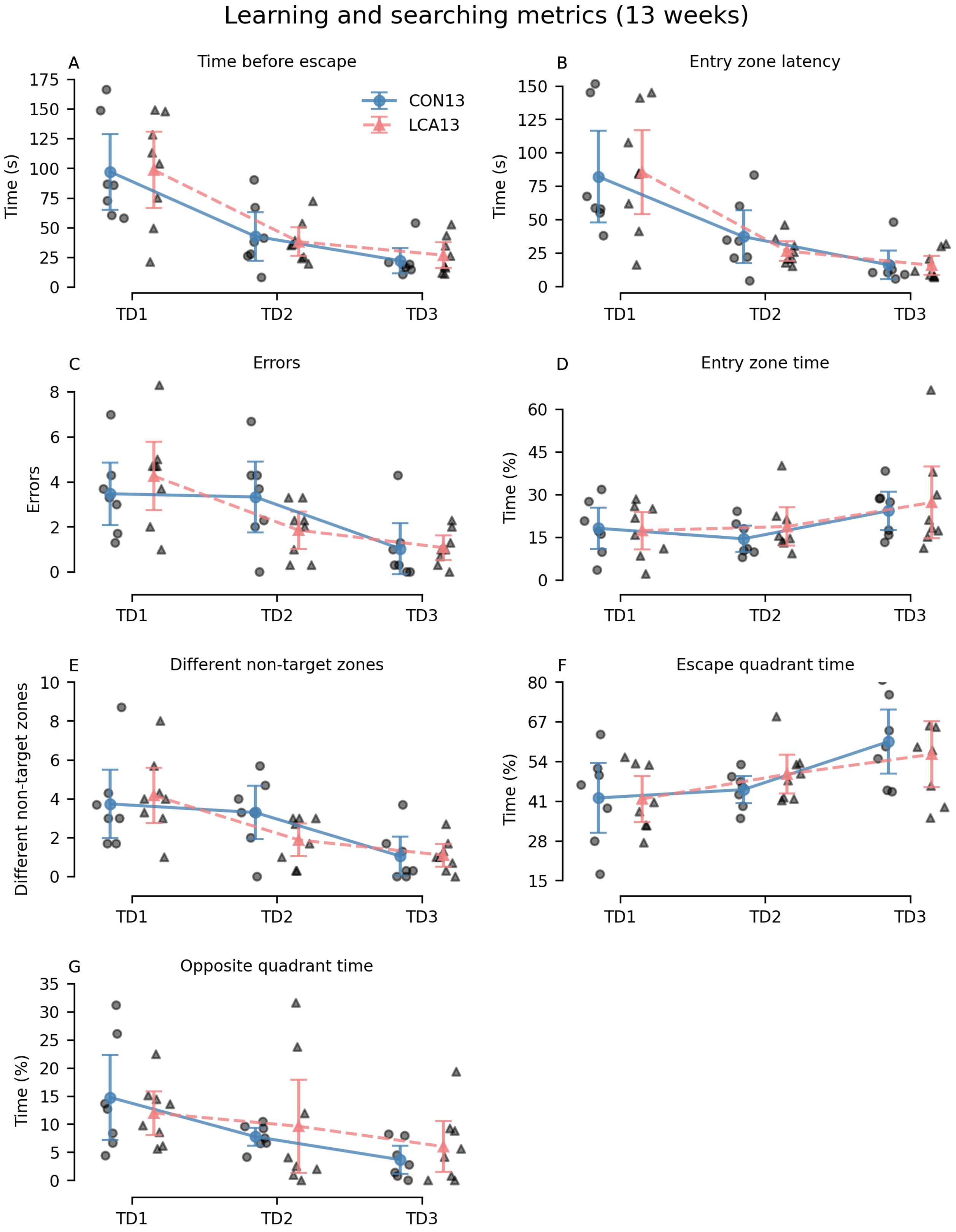

Supplementary Figure 9: The Barnes maze (BM) results of the 13-week-old mice during the training days (TDs). No statistically significant difference was observed between the groups.

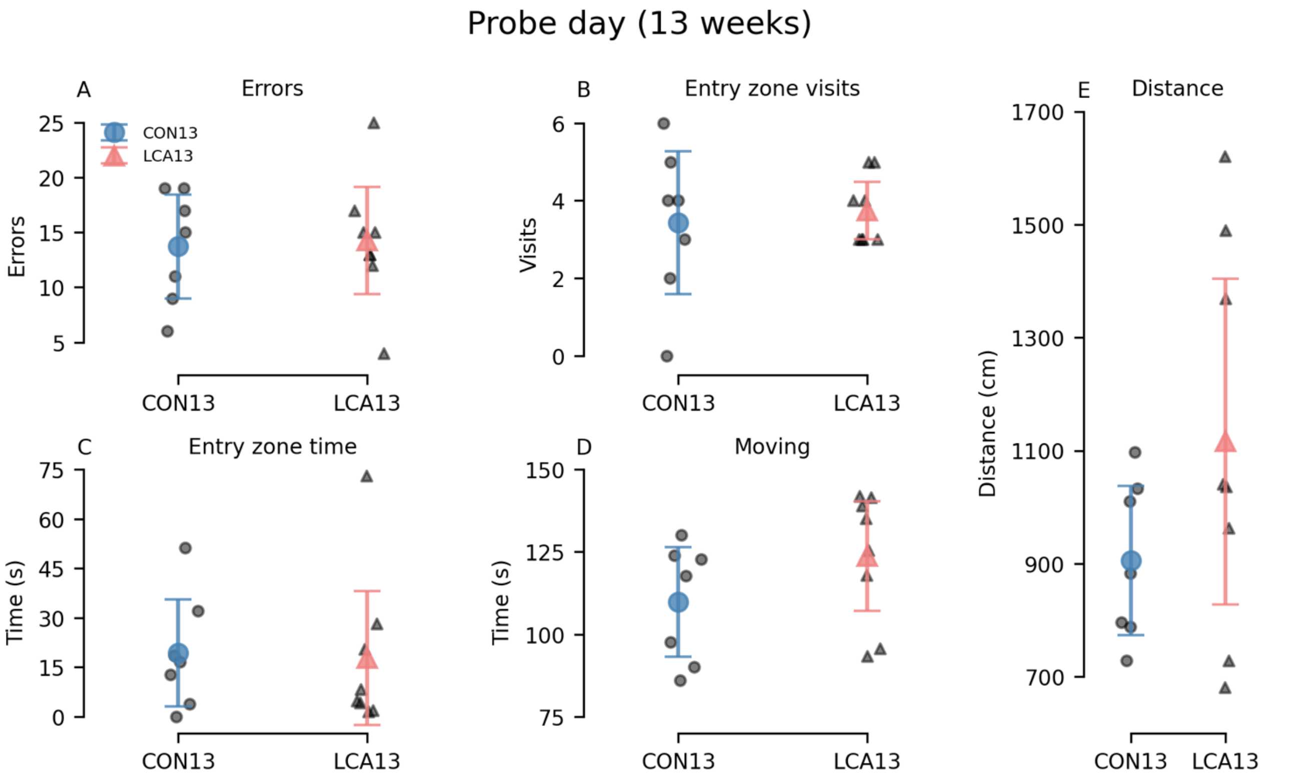

Supplementary Figure 10: The Barnes maze (BM) results of the 13-week-old mice during the probe day (PD). No statistically significant difference was observed between the groups.

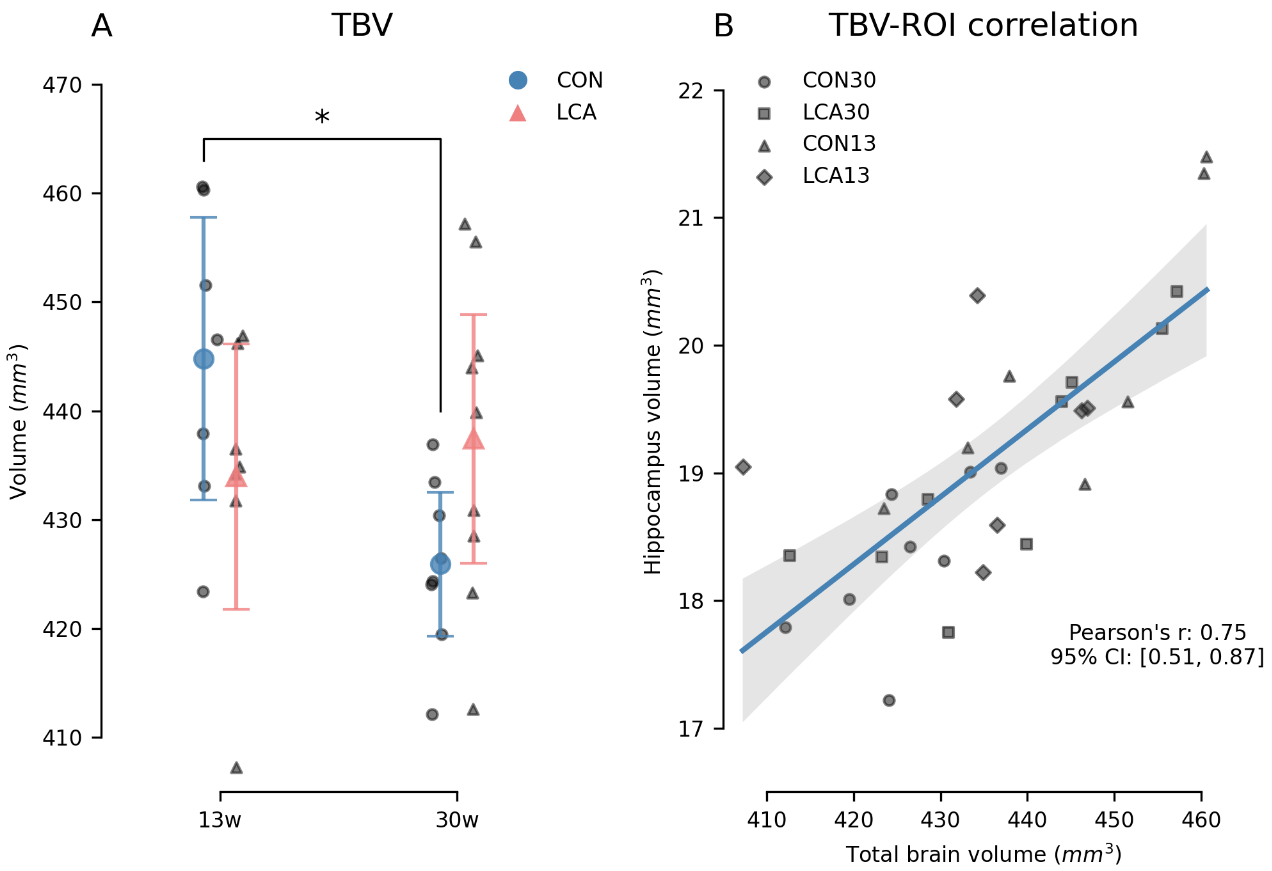

Supplementary Figure 11: (A) The total brain volume (TBV) of the four groups. No statistically significant difference was found between the treatments. However, the two CON groups differed. The group mean with 95% CI is superimposed on the individual observations. See table 10-11 for detailed descriptive statistics. * = Age difference in CON groups ($p < 0.05$). (B) Assessment of the linear correlation between TBV and the region of interest (ROI) hippocampus. The Pearson correlation coefficient (r) is plotted with its bootstrapped CI. The shaded field represents the 95% CI of the regression line. CON = control, LCA = LC-ablated. 13w = 13 weeks old, 30w = 30 weeks old.

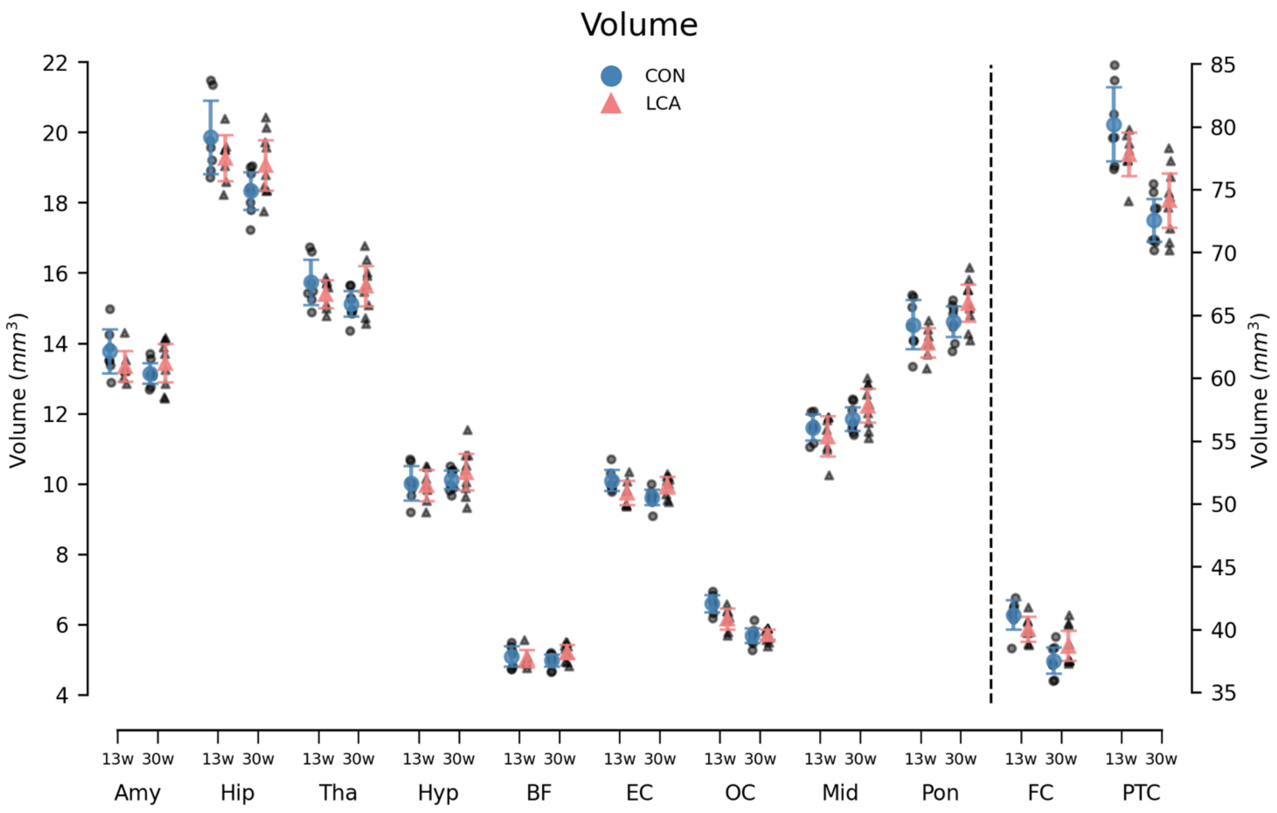

Supplementary Figure 12: The absolute ROI volumes. Note that FC and PTC belong to the right axis. See Table 10-11 for descriptive statistics. 13w = 13 weeks old, 30w = 30 weeks old. Amy = amygdala, hip = hippocampus, tha = thalamus, hyp = hypothalamus, BF = basal forebrain, EC = entorhinal cortex, OC = occipital cortex, mid = midbrain, pon = pons, FC = frontal cortex, PTC = parieto-temporal cortex.

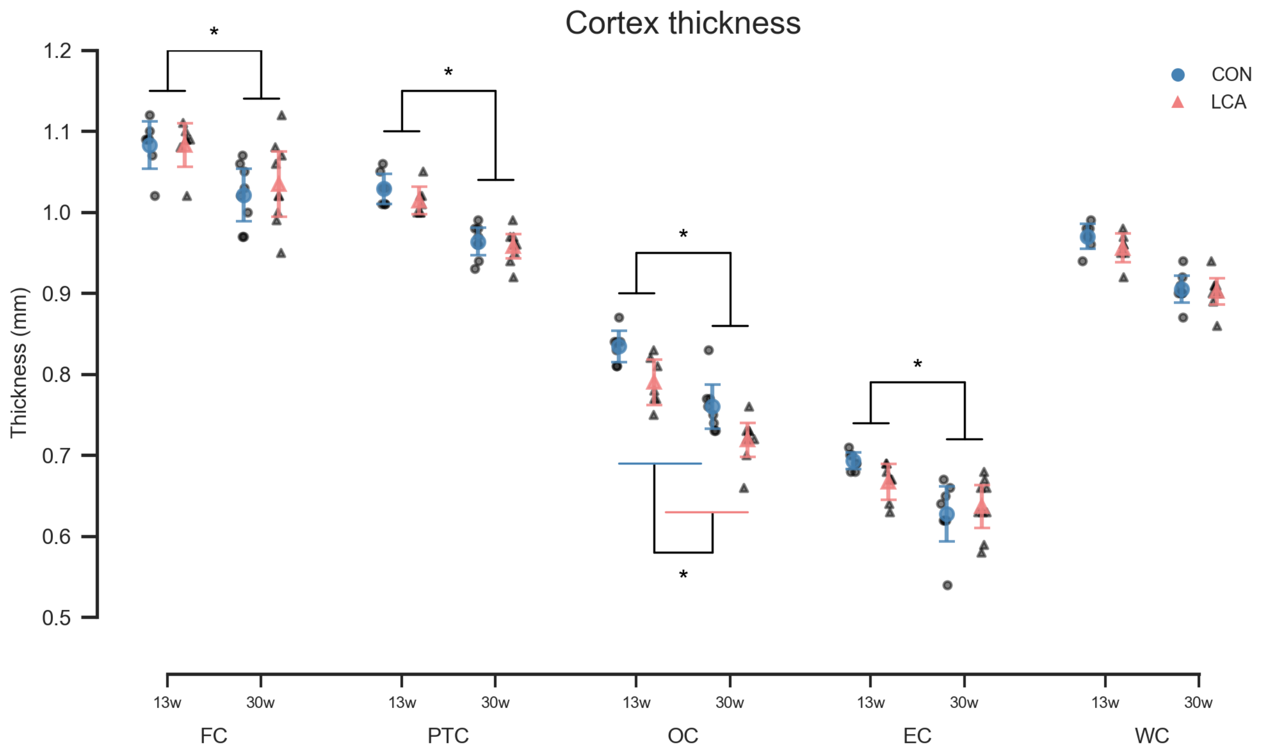

Supplementary Figure 13: The mean cortex thickness of four cortical regions and the mean of the entire cortex. See Supplementary Table 12 for descriptive statistics. 13w = 13 weeks old, 30w = 30 weeks old. FC = frontal cortex, PTC = parieto-temporal cortex, OC = occipital cortex, EC = entorhinal cortex, WC = whole cortex. The WC ROI was not included in the statistical model. Uncolored statistical markers indicate significant age effect. The colored statistical marker in OC indicates significant effect of treatment. * = $p < 0.05$.

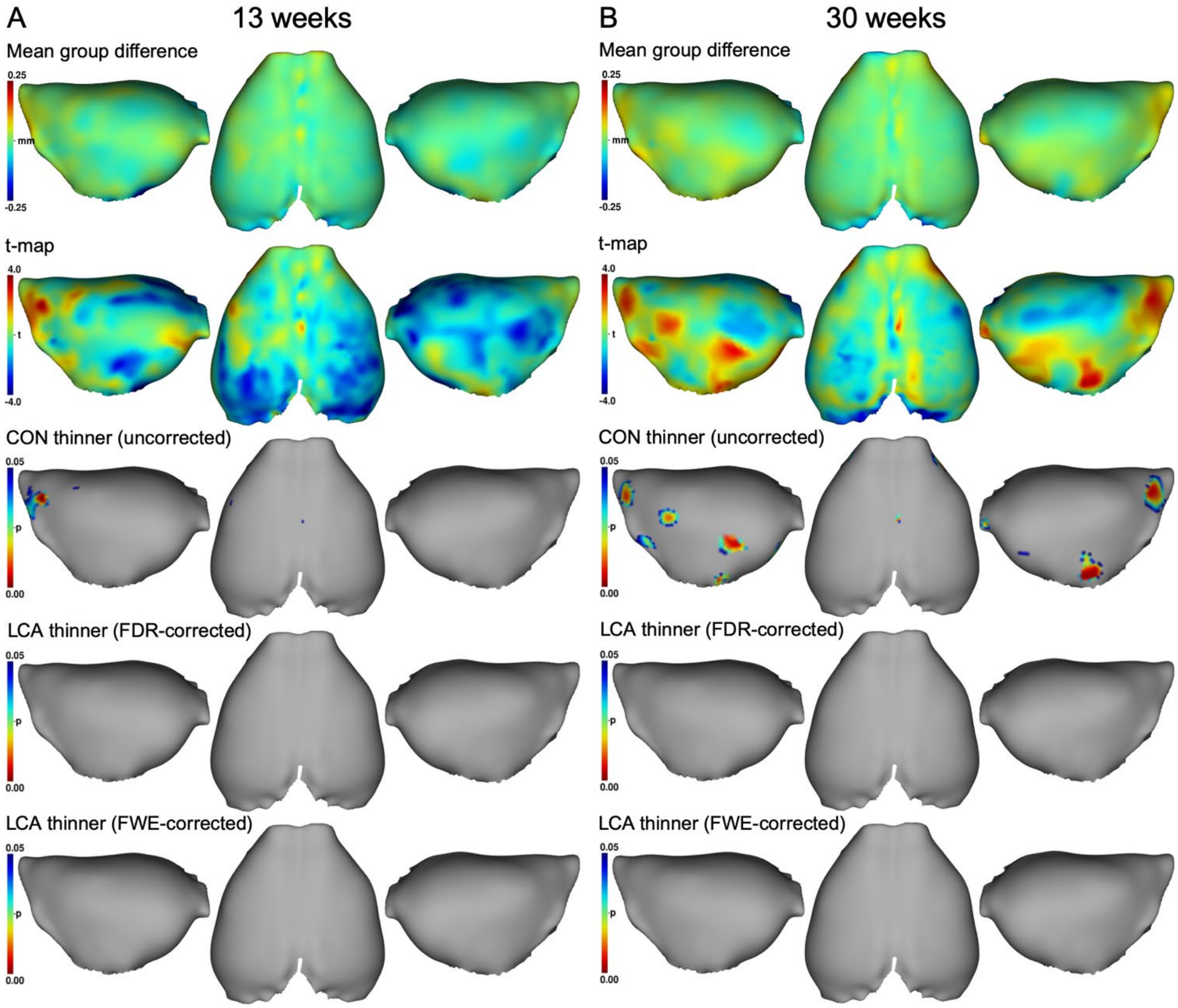

Supplementary Figure 14: Mean cortex thickness maps of **(A)** 13 weeks brains and **(B)** 30 weeks brains. First row: Difference maps between groups (CON minus LCA). Second row: t-maps of the group difference. Third row: p-maps of where the CON groups have thinner cortex, thresholded at 0.05 before correcting for multiple comparisons. Fourth and fifth row: Corrected p-maps of where the LCA groups have thinner cortex using either false discovery rate (FDR) or family-wise error rate (FEW), respectively.

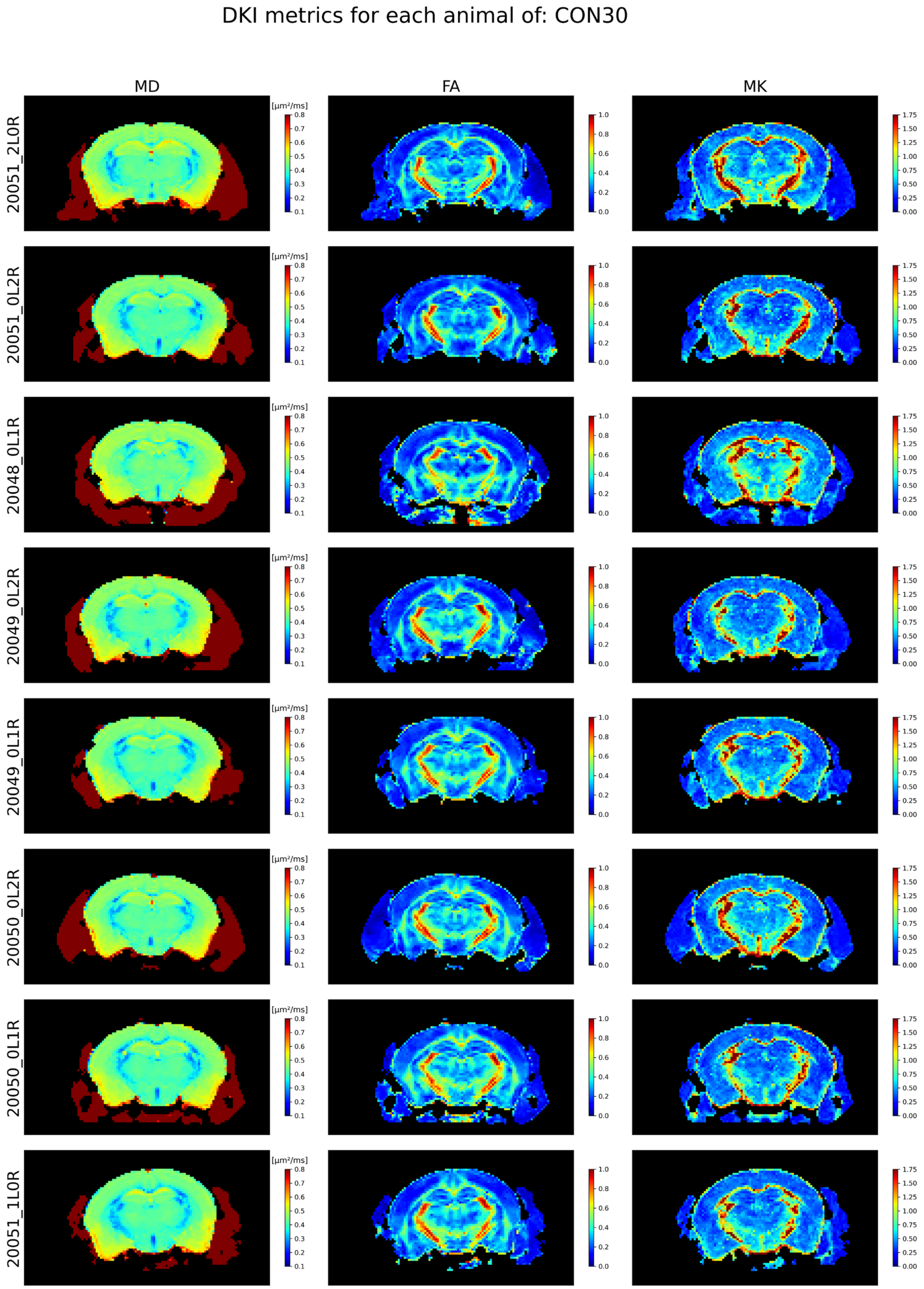

Supplementary Figure 15: CON30 group overview of calculated voxel-wise metrics from the DKI framework: mean diffusivity (MD; leftmost column), fractional anisotropy (FA; middle column) and mean kurtosis (MK; rightmost column) for the central coronal section of each subject.

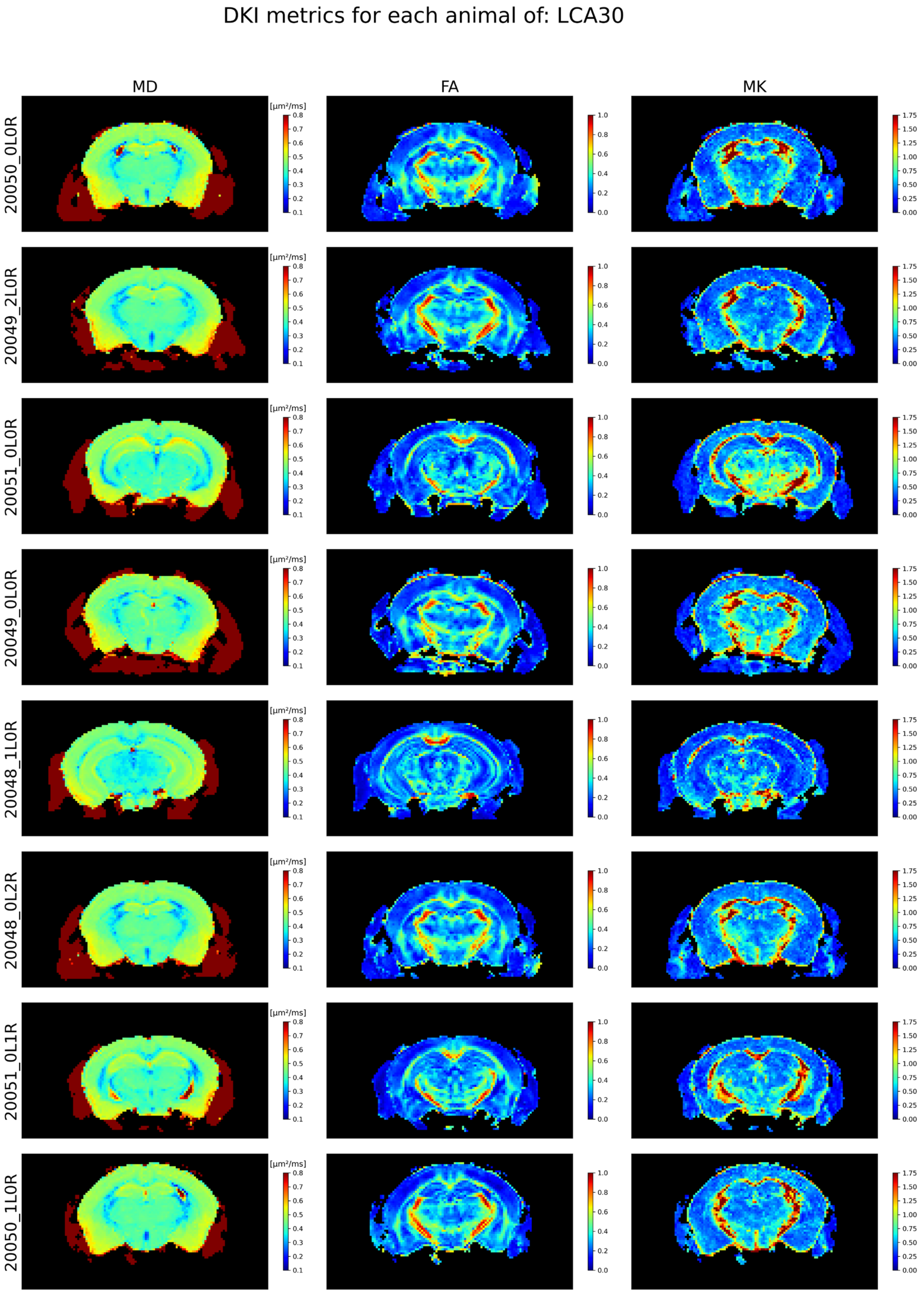

Supplementary Figure 16: LCA30 group overview of calculated voxel-wise metrics from the DKI framework: mean diffusivity (MD; leftmost column), fractional anisotropy (FA; middle column) and mean kurtosis (MK; rightmost column) for the central coronal section of each subject.

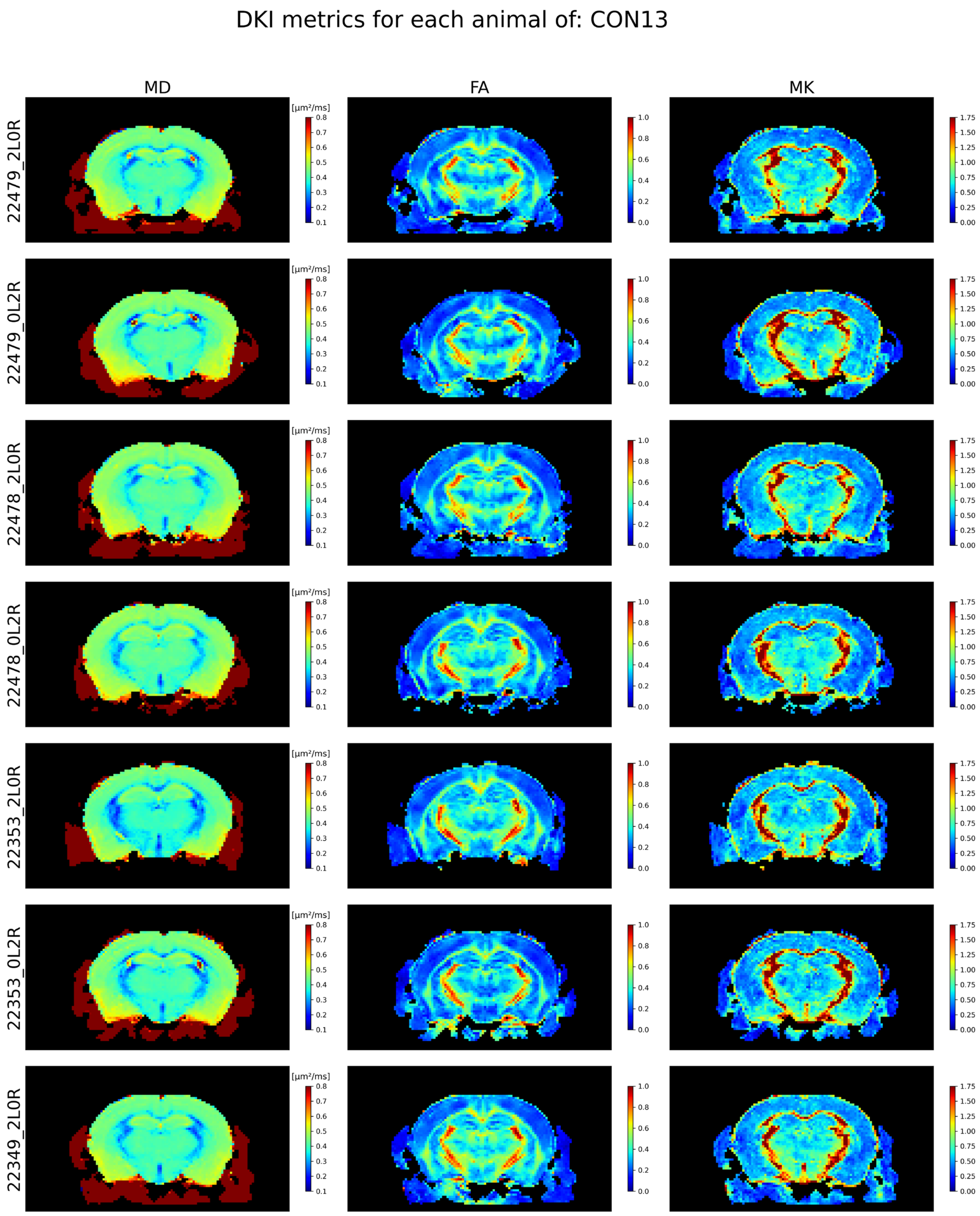

Supplementary Figure 17: CON13 group overview of calculated voxel-wise metrics from the DKI framework: mean diffusivity (MD; leftmost column), fractional anisotropy (FA; middle column) and mean kurtosis (MK; rightmost column) for the central coronal section of each subject.

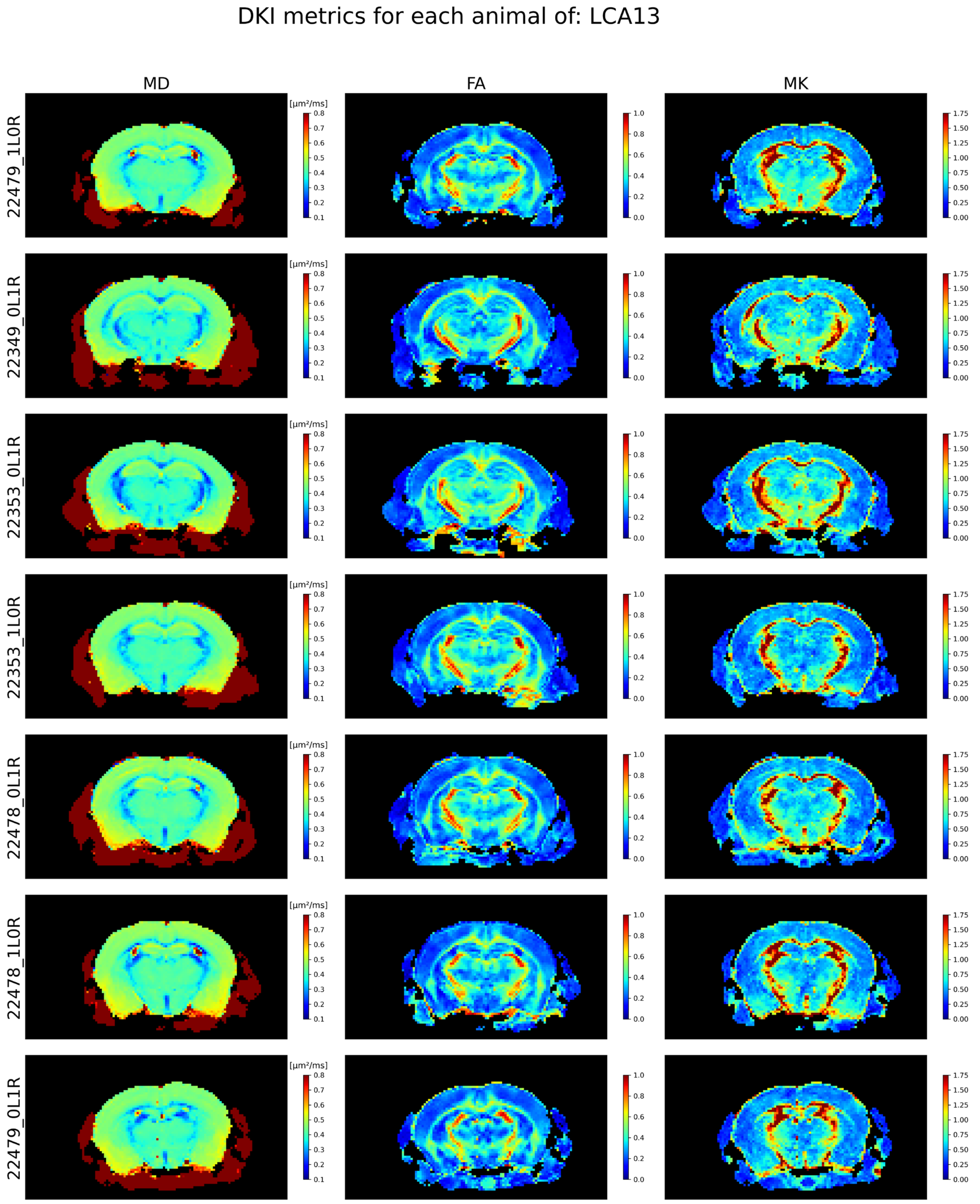

Supplementary Figure 18: LCA13 group overview of calculated voxel-wise metrics from the DKI framework: mean diffusivity (MD; leftmost column), fractional anisotropy (FA; middle column) and mean kurtosis (MK; rightmost column) for the central coronal section of each subject.

Supplementary Table 1: Descriptive statistics of the LDB test for the CON30/LCA30 groups. Lower = lower 95% CI, upper = upper 95% CI. Transitions = transitions from dark to light compartment.

Parameter	Time	Time bin	Group	n	Mean	Lower	Upper	Min	Max
Time in light	1 week post	First 5 min	CON30	10	42.3	30.5	54.1	2.9	69.7
Time in light	1 week post	First 5 min	LCA30	10	87.9	64.3	111.5	34.8	170.7
Time in light	1 week post	Last 5 min	CON30	10	87.8	67.0	108.5	20.6	133.6
Time in light	1 week post	Last 5 min	LCA30	10	113.6	81.3	145.9	55.8	203.1
Time in light	1 week pre	First 5 min	CON30	10	125.7	105.5	145.8	71.9	162.6
Time in light	1 week pre	First 5 min	LCA30	10	109.4	92.5	126.2	65.6	145.7
Time in light	1 week pre	Last 5 min	CON30	10	149.5	119.4	179.7	69.9	222.1
Time in light	1 week pre	Last 5 min	LCA30	10	111.4	90.8	132.1	63.9	162.1
Time in light	8 weeks post	First 5 min	CON30	10	41.9	24.2	59.6	7.5	81.6
Time in light	8 weeks post	First 5 min	LCA30	10	63.5	32.3	94.7	9.0	141.7
Time in light	8 weeks post	Last 5 min	CON30	10	119.7	71.2	168.2	26.8	231.5
Time in light	8 weeks post	Last 5 min	LCA30	10	117.6	77.2	157.9	6.5	215.0
Time along walls	1 week post	First 5 min	CON30	10	86.9	81.9	91.9	72.3	95.4
Time along walls	1 week post	First 5 min	LCA30	10	85.8	80.7	90.9	72.4	95.3
Time along walls	1 week post	Last 5 min	CON30	10	84.8	77.4	92.2	65.3	99.0
Time along walls	1 week post	Last 5 min	LCA30	10	84.3	78.2	90.4	69.2	94.3
Time along walls	1 week pre	First 5 min	CON30	10	76.3	71.5	81.1	67.7	90.7
Time along walls	1 week pre	First 5 min	LCA30	10	80.0	74.0	85.9	60.7	93.0
Time along walls	1 week pre	Last 5 min	CON30	10	75.1	64.7	85.6	43.0	98.1
Time along walls	1 week pre	Last 5 min	LCA30	10	85.3	80.0	90.5	68.8	94.1
Time along walls	8 weeks post	First 5 min	CON30	10	85.2	76.8	93.6	59.9	98.6
Time along walls	8 weeks post	First 5 min	LCA30	10	89.0	81.9	96.1	60.5	99.3
Time along walls	8 weeks post	Last 5 min	CON30	10	80.2	68.9	91.4	42.0	98.2
Time along walls	8 weeks post	Last 5 min	LCA30	10	87.4	78.7	96.1	55.3	100.0
Transitions	1 week post	First 5 min	CON30	10	3.9	2.4	5.4	0.0	8.0
Transitions	1 week post	First 5 min	LCA30	10	5.4	3.5	7.3	3.0	10.0
Transitions	1 week post	Last 5 min	CON30	10	5.2	3.9	6.5	1.0	8.0
Transitions	1 week post	Last 5 min	LCA30	10	4.9	2.9	6.9	2.0	12.0
Transitions	1 week pre	First 5 min	CON30	10	9.8	8.4	11.2	6.0	14.0
Transitions	1 week pre	First 5 min	LCA30	10	9.3	8.1	10.5	7.0	13.0
Transitions	1 week pre	Last 5 min	CON30	10	8.7	6.8	10.6	5.0	14.0
Transitions	1 week pre	Last 5 min	LCA30	10	6.3	5.3	7.3	4.0	9.0
Transitions	8 weeks post	First 5 min	CON30	10	2.9	1.7	4.1	1.0	7.0
Transitions	8 weeks post	First 5 min	LCA30	10	3.6	1.6	5.6	1.0	11.0
Transitions	8 weeks post	Last 5 min	CON30	10	5.0	3.1	6.9	0.0	9.0
Transitions	8 weeks post	Last 5 min	LCA30	10	4.2	2.5	5.9	0.0	9.0

Supplementary Table 2: Descriptive statistics of the LDB test for the CON30/LCA30 groups. Lower = lower 95% CI, upper = upper 95% CI. Transitions = transitions from dark to light compartment.

Parameter	Time	Group	n	Mean	Lower	Upper	Min	Max
Latency to first light-dark transition	1 week post	CON30	10	7.0	3.5	10.6	1.8	18.5
Latency to first light-dark transition	1 week post	LCA30	10	4.4	2.4	6.5	1.1	10.2
Latency to first light-dark transition	1 week pre	CON30	10	30.1	22.1	38.1	13.6	51.1
Latency to first light-dark transition	1 week pre	LCA30	10	28.6	14.5	42.6	5.6	75.5

Parameter	Time	Group	n	Mean	Lower	Upper	Min	Max
Latency to first light-dark transition	8 weeks post	CON30	10	4.0	2.0	6.1	1.6	11.9
Latency to first light-dark transition	8 weeks post	LCA30	10	4.4	2.2	6.6	1.2	11.1
Time in dark before first transition	1 week post	CON30	10	97.5	30.2	164.9	7.1	297.4
Time in dark before first transition	1 week post	LCA30	10	71.9	36.1	107.7	6.0	186.1
Time in dark before first transition	1 week pre	CON30	10	13.7	10.2	17.1	6.8	25.6
Time in dark before first transition	1 week pre	LCA30	10	16.6	9.9	23.3	6.4	35.9
Time in dark before first transition	8 weeks post	CON30	10	96.5	50.1	142.9	9.9	247.7
Time in dark before first transition	8 weeks post	LCA30	10	85.8	35.2	136.3	8.4	227.3

Supplementary Table 3: Descriptive statistics of the LDB test for the CON13/LCA13 groups. Lower = lower 95% CI, upper = upper 95% CI. Transitions = transitions from dark to light compartment.

Parameter	Time bin	Group	n	Mean	Lower	Upper	Min	Max
Time in light	First 5 min	CON13	8	121.4	108.1	134.8	93.5	141.8
Time in light	First 5 min	LCA13	7	116.6	98.5	134.7	68.0	144.0
Time in light	Last 5 min	CON13	8	133.3	99.0	167.5	43.7	198.9
Time in light	Last 5 min	LCA13	7	169.5	147.5	191.5	139.3	213.5
Time along walls	First 5 min	CON13	8	72.9	67.9	77.9	64.5	85.2
Time along walls	First 5 min	LCA13	7	80.1	75.1	85.0	70.8	86.9
Time along walls	Last 5 min	CON13	8	66.2	58.9	73.4	46.0	76.4
Time along walls	Last 5 min	LCA13	7	69.6	61.2	77.9	57.2	86.2
Transitions	First 5 min	CON13	8	8.0	6.4	9.6	5.0	12.0
Transitions	First 5 min	LCA13	7	8.6	7.5	9.7	6.0	10.0
Transitions	Last 5 min	CON13	8	5.6	4.6	6.7	3.0	8.0
Transitions	Last 5 min	LCA13	7	7.0	5.5	8.5	4.0	9.0

Supplementary Table 4: Descriptive statistics of the LDB test for the CON13/LCA13 groups. Lower = lower 95% CI, upper = upper 95% CI. Transitions = transitions from dark to light compartment.

Parameter	Group	n	Mean	Lower	Upper	Min	Max
Latency to first light-dark transition	CON13	8	25.2	9.5	41.0	11.5	79.9
Latency to first light-dark transition	LCA13	7	23.5	9.3	37.6	9.2	64.3
Time in dark before first transition	CON13	8	16.6	11.6	21.6	8.7	29.0
Time in dark before first transition	LCA13	7	17.5	13.4	21.6	12.9	28.0

Supplementary Table 5: Descriptive statistics of the BM test for the CON30/LCA30 groups. Lower = lower 95% CI, upper = upper 95% CI.

Parameter	Time	Day	Group	n	Mean	Lower	Upper	Min	Max
Different non-target zones	1 week post	TD1	CON30	10	1.8	0.8	2.8	0.3	5.0
Different non-target zones	1 week post	TD1	LCA30	10	1.8	0.7	3.0	0.0	4.7
Different non-target zones	1 week post	TD2	CON30	10	0.8	0.1	1.4	0.0	3.0
Different non-target zones	1 week post	TD2	LCA30	10	0.4	0.1	0.7	0.0	1.3
Different non-target zones	1 week post	TD3	CON30	10	0.3	-0.1	0.7	0.0	2.0

Parameter	Time	Day	Group	n	Mean	Lower	Upper	Min	Max
Different non-target zones	1 week post	TD3	LCA30	10	1.1	0.2	2.1	0.0	4.7
Different non-target zones	1 week pre	TD1	CON30	10	4.7	3.5	5.8	1.3	6.7
Different non-target zones	1 week pre	TD1	LCA30	10	3.0	1.6	4.4	0.3	8.0
Different non-target zones	1 week pre	TD2	CON30	10	2.3	1.4	3.2	0.3	4.7
Different non-target zones	1 week pre	TD2	LCA30	10	1.3	0.7	1.9	0.3	3.3
Different non-target zones	1 week pre	TD3	CON30	10	1.8	0.9	2.7	0.0	4.7
Different non-target zones	1 week pre	TD3	LCA30	10	1.7	0.5	2.9	0.0	5.3
Different non-target zones	8 weeks post	TD1	CON30	10	1.2	0.4	1.9	0.3	3.7
Different non-target zones	8 weeks post	TD1	LCA30	10	2.9	1.5	4.4	0.0	6.7
Different non-target zones	8 weeks post	TD2	CON30	10	0.7	0.1	1.4	0.0	2.7
Different non-target zones	8 weeks post	TD2	LCA30	10	0.4	0.1	0.7	0.0	1.3
Different non-target zones	8 weeks post	TD3	CON30	10	0.4	0.1	0.7	0.0	1.7
Different non-target zones	8 weeks post	TD3	LCA30	10	0.9	0.2	1.6	0.0	2.7
Entry zone latency	1 week post	TD1	CON30	10	26.2	4.9	47.6	6.2	122.8
Entry zone latency	1 week post	TD1	LCA30	10	34.2	7.1	61.3	2.3	133.5
Entry zone latency	1 week post	TD2	CON30	10	25.6	9.2	42.0	4.6	89.4
Entry zone latency	1 week post	TD2	LCA30	10	12.5	1.5	23.6	1.5	62.0
Entry zone latency	1 week post	TD3	CON30	10	10.8	2.3	19.4	2.8	42.8
Entry zone latency	1 week post	TD3	LCA30	10	21.0	7.2	34.8	2.0	66.0
Entry zone latency	1 week pre	TD1	CON30	10	77.9	47.6	108.2	20.6	164.3
Entry zone latency	1 week pre	TD1	LCA30	10	49.0	20.7	77.2	5.5	123.7
Entry zone latency	1 week pre	TD2	CON30	10	60.2	14.2	106.2	6.8	180.0
Entry zone latency	1 week pre	TD2	LCA30	10	26.6	13.1	40.2	5.5	68.1
Entry zone latency	1 week pre	TD3	CON30	10	36.5	12.4	60.7	3.3	118.3
Entry zone latency	1 week pre	TD3	LCA30	10	20.7	5.7	35.8	6.0	87.6
Entry zone latency	8 weeks post	TD1	CON30	10	20.4	9.2	31.5	6.2	66.2
Entry zone latency	8 weeks post	TD1	LCA30	10	27.8	8.3	47.3	7.0	109.7
Entry zone latency	8 weeks post	TD2	CON30	10	10.6	2.7	18.5	2.3	45.9
Entry zone latency	8 weeks post	TD2	LCA30	10	10.2	1.9	18.4	2.8	47.6
Entry zone latency	8 weeks post	TD3	CON30	10	6.6	4.0	9.2	2.6	14.5
Entry zone latency	8 weeks post	TD3	LCA30	10	7.7	5.6	9.8	2.5	11.9

Supplementary Table 6: Descriptive statistics of the BM test for the CON30/LCA30 groups. Lower = lower 95% CI, upper = upper 95% CI.

Parameter	Time	Day	Group	n	Mean	Lower	Upper	Min	Max
Entry zone time	1 week post	TD1	CON30	10	17.0	11.5	22.5	6.3	32.0
Entry zone time	1 week post	TD1	LCA30	10	20.0	11.8	28.1	3.5	41.9
Entry zone time	1 week post	TD2	CON30	10	21.0	13.7	28.3	8.7	47.7
Entry zone time	1 week post	TD2	LCA30	10	27.1	17.1	37.0	11.0	59.7
Entry zone time	1 week post	TD3	CON30	10	29.9	18.3	41.5	11.9	75.8
Entry zone time	1 week post	TD3	LCA30	10	22.9	14.6	31.2	1.6	42.7
Entry zone time	1 week pre	TD1	CON30	10	29.4	16.8	42.0	5.1	64.1
Entry zone time	1 week pre	TD1	LCA30	10	36.1	21.5	50.7	8.7	79.7
Entry zone time	1 week pre	TD2	CON30	10	28.4	13.2	43.7	0.0	69.7
Entry zone time	1 week pre	TD2	LCA30	10	29.5	21.2	37.8	8.5	48.5
Entry zone time	1 week pre	TD3	CON30	10	26.9	18.0	35.8	8.1	50.9

Parameter	Time	Day	Group	n	Mean	Lower	Upper	Min	Max
Entry zone time	1 week pre	TD3	LCA30	10	28.3	18.7	37.9	7.9	53.3
Entry zone time	8 weeks post	TD1	CON30	10	22.5	16.0	29.0	7.6	38.5
Entry zone time	8 weeks post	TD1	LCA30	10	19.3	11.9	26.7	3.8	40.3
Entry zone time	8 weeks post	TD2	CON30	10	29.7	22.2	37.2	13.7	44.7
Entry zone time	8 weeks post	TD2	LCA30	10	21.9	16.1	27.6	9.6	38.3
Entry zone time	8 weeks post	TD3	CON30	10	27.1	22.0	32.2	15.3	36.9
Entry zone time	8 weeks post	TD3	LCA30	10	23.8	16.8	30.8	11.4	39.9
Errors	1 week post	TD1	CON30	10	1.9	0.9	3.0	0.3	5.3
Errors	1 week post	TD1	LCA30	10	2.1	0.8	3.4	0.0	5.3
Errors	1 week post	TD2	CON30	10	1.1	0.1	2.2	0.0	5.0
Errors	1 week post	TD2	LCA30	10	0.4	0.1	0.7	0.0	1.3
Errors	1 week post	TD3	CON30	10	0.3	-0.1	0.7	0.0	2.0
Errors	1 week post	TD3	LCA30	10	1.4	0.1	2.7	0.0	6.3
Errors	1 week pre	TD1	CON30	10	6.2	4.4	7.9	1.3	11.3
Errors	1 week pre	TD1	LCA30	10	3.4	1.4	5.3	0.3	11.0
Errors	1 week pre	TD2	CON30	10	2.5	1.5	3.5	0.3	5.0
Errors	1 week pre	TD2	LCA30	10	1.5	0.8	2.2	0.3	3.7
Errors	1 week pre	TD3	CON30	10	2.1	0.9	3.4	0.0	6.3
Errors	1 week pre	TD3	LCA30	10	2.0	0.6	3.4	0.0	6.0
Errors	8 weeks post	TD1	CON30	10	1.3	0.4	2.3	0.3	5.0
Errors	8 weeks post	TD1	LCA30	10	3.3	1.6	5.1	0.0	9.0
Errors	8 weeks post	TD2	CON30	10	0.8	0.1	1.5	0.0	3.3
Errors	8 weeks post	TD2	LCA30	10	0.4	0.1	0.7	0.0	1.3
Errors	8 weeks post	TD3	CON30	10	0.5	0.1	0.9	0.0	2.0
Errors	8 weeks post	TD3	LCA30	10	0.9	0.2	1.6	0.0	2.7

Supplementary Table 7: Descriptive statistics of the BM test for the CON30/LCA30 groups. Lower = lower 95% CI, upper = upper 95% CI.

Parameter	Time	Day	Group	n	Mean	Lower	Upper	Min	Max
Escape quadrant time	1 week post	TD1	CON30	10	55.7	46.6	64.7	32.5	74.8
Escape quadrant time	1 week post	TD1	LCA30	10	55.1	47.9	62.4	39.9	76.7
Escape quadrant time	1 week post	TD2	CON30	10	62.4	53.2	71.5	44.0	86.2
Escape quadrant time	1 week post	TD2	LCA30	10	59.3	48.2	70.4	35.8	84.0
Escape quadrant time	1 week post	TD3	CON30	10	62.9	54.0	71.9	41.6	90.3
Escape quadrant time	1 week post	TD3	LCA30	10	58.9	51.0	66.7	32.6	77.8
Escape quadrant time	1 week pre	TD1	CON30	10	51.4	37.4	65.4	19.3	90.8
Escape quadrant time	1 week pre	TD1	LCA30	10	63.2	51.4	75.0	25.6	91.2
Escape quadrant time	1 week pre	TD2	CON30	10	51.4	35.5	67.3	15.9	86.8
Escape quadrant time	1 week pre	TD2	LCA30	10	58.3	49.5	67.0	31.7	75.4
Escape quadrant time	1 week pre	TD3	CON30	10	63.8	58.1	69.4	48.3	76.4
Escape quadrant time	1 week pre	TD3	LCA30	10	61.1	48.0	74.2	24.8	87.3
Escape quadrant time	8 weeks post	TD1	CON30	10	55.9	47.7	64.1	30.5	77.6
Escape quadrant time	8 weeks post	TD1	LCA30	10	49.4	41.6	57.1	30.3	65.7
Escape quadrant time	8 weeks post	TD2	CON30	10	56.7	49.3	64.1	40.5	79.5
Escape quadrant time	8 weeks post	TD2	LCA30	10	53.8	47.7	59.9	38.4	71.1
Escape quadrant time	8 weeks post	TD3	CON30	10	58.7	53.7	63.6	41.1	69.3

Parameter	Time	Day	Group	n	Mean	Lower	Upper	Min	Max
Escape quadrant time	8 weeks post	TD3	LCA30	10	52.2	44.6	59.8	36.6	69.7
Opposite quadrant time	1 week post	TD1	CON30	10	9.7	2.2	17.2	0.0	40.7
Opposite quadrant time	1 week post	TD1	LCA30	10	6.9	2.7	11.1	0.0	21.8
Opposite quadrant time	1 week post	TD2	CON30	10	4.8	1.1	8.4	0.0	15.2
Opposite quadrant time	1 week post	TD2	LCA30	10	6.1	0.3	11.9	0.0	31.4
Opposite quadrant time	1 week post	TD3	CON30	10	0.5	0.1	0.8	0.0	1.4
Opposite quadrant time	1 week post	TD3	LCA30	10	2.6	1.0	4.1	0.0	6.4
Opposite quadrant time	1 week pre	TD1	CON30	10	9.5	2.6	16.4	0.0	33.1
Opposite quadrant time	1 week pre	TD1	LCA30	10	8.0	1.0	15.0	0.0	34.8
Opposite quadrant time	1 week pre	TD2	CON30	10	5.8	1.6	10.0	0.0	18.3
Opposite quadrant time	1 week pre	TD2	LCA30	10	10.3	4.4	16.3	0.0	31.7
Opposite quadrant time	1 week pre	TD3	CON30	10	2.5	0.1	4.9	0.0	9.5
Opposite quadrant time	1 week pre	TD3	LCA30	10	6.3	0.0	12.7	0.0	31.3
Opposite quadrant time	8 weeks post	TD1	CON30	10	8.4	4.4	12.4	0.4	19.5
Opposite quadrant time	8 weeks post	TD1	LCA30	10	15.4	10.1	20.6	1.4	26.7
Opposite quadrant time	8 weeks post	TD2	CON30	10	5.4	0.5	10.4	0.0	22.3
Opposite quadrant time	8 weeks post	TD2	LCA30	10	9.1	3.7	14.6	0.0	24.9
Opposite quadrant time	8 weeks post	TD3	CON30	10	4.5	0.5	8.5	0.0	18.1
Opposite quadrant time	8 weeks post	TD3	LCA30	10	4.6	1.1	8.1	0.0	14.5

Supplementary Table 8: Descriptive statistics of the BM test for the CON30/LCA30 groups. Lower = lower 95% CI, upper = upper 95% CI.

Parameter	Time	Day	Group	n	Mean	Lower	Upper	Min	Max
Time before escape	1 week post	TD1	CON30	10	33.7	13.5	53.9	18.1	124.9
Time before escape	1 week post	TD1	LCA30	10	40.7	12.9	68.4	6.4	139.1
Time before escape	1 week post	TD2	CON30	10	31.9	15.7	48.1	7.9	92.4
Time before escape	1 week post	TD2	LCA30	10	19.7	7.6	31.7	4.9	73.4
Time before escape	1 week post	TD3	CON30	10	19.3	9.3	29.3	7.2	51.3
Time before escape	1 week post	TD3	LCA30	10	27.9	13.5	42.4	5.4	69.6
Time before escape	1 week pre	TD1	CON30	10	119.5	94.1	145.0	65.5	180.0
Time before escape	1 week pre	TD1	LCA30	10	88.0	54.4	121.6	37.3	179.9
Time before escape	1 week pre	TD2	CON30	10	83.4	41.8	124.9	17.3	179.9
Time before escape	1 week pre	TD2	LCA30	10	48.0	24.2	71.8	9.5	134.9
Time before escape	1 week pre	TD3	CON30	10	50.9	22.7	79.0	11.2	153.0
Time before escape	1 week pre	TD3	LCA30	10	31.4	12.0	50.9	12.1	119.4
Time before escape	8 weeks post	TD1	CON30	10	29.8	17.4	42.1	11.7	68.6
Time before escape	8 weeks post	TD1	LCA30	10	40.6	20.5	60.8	11.5	117.6
Time before escape	8 weeks post	TD2	CON30	10	19.8	10.7	28.8	5.7	52.3
Time before escape	8 weeks post	TD2	LCA30	10	16.4	7.3	25.4	7.2	55.3
Time before escape	8 weeks post	TD3	CON30	10	12.5	9.2	15.8	6.0	22.6
Time before escape	8 weeks post	TD3	LCA30	10	13.2	10.1	16.3	7.6	22.1

Supplementary Table 9: Parameter: Relative volume as percentage of TBV. Amy = amygdala, hip = hippocampus, tha = thalamus, hyp = hypothalamus, BF = basal forebrain, EC = entorhinal cortex, OC

= occipital cortex, mid = midbrain, pon = pons, FC = frontal cortex, PTC = parieto-temporal cortex. Lower = lower 95% CI, upper = upper 95% CI.

ROI	Group	n	Mean	Lower	Upper	Min	Max
Amy	CON13	7	3.13	3.13	3.13	3.13	3.14
Amy	CON30	9	3.13	3.12	3.14	3.12	3.15
Amy	LCA13	7	3.13	3.13	3.13	3.13	3.13
Amy	LCA30	9	3.13	3.13	3.13	3.13	3.14
Hip	CON13	7	4.58	4.58	4.58	4.58	4.59
Hip	CON30	9	4.58	4.57	4.59	4.57	4.60
Hip	LCA13	7	4.58	4.58	4.58	4.57	4.58
Hip	LCA30	9	4.58	4.57	4.58	4.57	4.59
Tha	CON13	7	3.59	3.58	3.59	3.58	3.60
Tha	CON30	9	3.59	3.58	3.60	3.58	3.60
Tha	LCA13	7	3.59	3.58	3.59	3.58	3.60
Tha	LCA30	9	3.59	3.58	3.59	3.57	3.60
Hyp	CON13	7	2.41	2.40	2.41	2.40	2.42
Hyp	CON30	9	2.41	2.40	2.42	2.38	2.43
Hyp	LCA13	7	2.40	2.40	2.41	2.39	2.42
Hyp	LCA30	9	2.42	2.40	2.43	2.39	2.44
BF	CON13	7	1.10	1.09	1.10	1.09	1.10
BF	CON30	9	1.10	1.09	1.10	1.09	1.10
BF	LCA13	7	1.09	1.09	1.10	1.09	1.10
BF	LCA30	9	1.10	1.09	1.10	1.09	1.10
EC	CON13	7	2.31	2.31	2.31	2.31	2.31
EC	CON30	9	2.31	2.31	2.31	2.30	2.32
EC	LCA13	7	2.31	2.31	2.31	2.30	2.31
EC	LCA30	9	2.31	2.31	2.31	2.30	2.32
FC	CON13	7	9.45	9.43	9.46	9.43	9.48
FC	CON30	9	9.43	9.42	9.45	9.41	9.49
FC	LCA13	7	9.44	9.43	9.45	9.42	9.46
FC	LCA30	9	9.43	9.42	9.45	9.41	9.47
OC	CON13	7	1.45	1.45	1.45	1.45	1.45
OC	CON30	9	1.45	1.45	1.45	1.45	1.46
OC	LCA13	7	1.45	1.45	1.45	1.45	1.45
OC	LCA30	9	1.45	1.45	1.45	1.45	1.46
PTC	CON13	7	17.59	17.58	17.61	17.57	17.63
PTC	CON30	9	17.57	17.55	17.60	17.55	17.66
PTC	LCA13	7	17.57	17.55	17.58	17.55	17.59
PTC	LCA30	9	17.57	17.56	17.59	17.55	17.61

Supplementary Table 10: Parameter: Absolute volume of ROIs provided in mm^3. Amy = amygdala, hip = hippocampus, tha = thalamus, hyp = hypothalamus, BF = basal forebrain, EC = entorhinal cortex, OC = occipital cortex, mid = midbrain, pon = pons, FC = frontal cortex, PTC = parieto-temporal cortex. Lower = lower 95% CI, upper = upper 95% CI.

ROI	Group	n	Mean	Lower	Upper	Min	Max
Amy	CON13	7	13.77	13.27	14.27	12.88	14.97

ROI	Group	n	Mean	Lower	Upper	Min	Max
Amy	CON30	8	13.14	12.90	13.38	12.68	13.70
Amy	LCA13	7	13.34	12.99	13.69	12.84	14.30
Amy	LCA30	9	13.43	12.97	13.90	12.43	14.16
BF	CON13	7	5.08	4.85	5.31	4.73	5.48
BF	CON30	8	4.97	4.83	5.12	4.65	5.20
BF	LCA13	7	5.03	4.84	5.22	4.76	5.57
BF	LCA30	9	5.21	5.04	5.39	4.81	5.51
EC	CON13	7	10.09	9.85	10.33	9.77	10.71
EC	CON30	8	9.61	9.43	9.79	9.09	10.00
EC	LCA13	7	9.74	9.46	10.02	9.35	10.34
EC	LCA30	9	9.96	9.76	10.16	9.48	10.29
FC	CON13	7	41.14	40.20	42.09	38.53	42.54
FC	CON30	8	37.51	36.64	38.38	35.90	39.44
FC	LCA13	7	40.02	39.23	40.81	38.76	41.75
FC	LCA30	9	38.68	37.66	39.70	37.28	41.14
OC	CON13	7	6.59	6.39	6.79	6.18	6.95
OC	CON30	8	5.68	5.50	5.86	5.27	6.12
OC	LCA13	7	6.16	5.92	6.40	5.69	6.57
OC	LCA30	9	5.70	5.58	5.83	5.38	5.89
PTC	CON13	7	80.20	77.84	82.56	76.65	84.92
PTC	CON30	8	72.54	71.13	73.95	70.19	75.48
PTC	LCA13	7	77.81	76.41	79.20	74.11	79.80
PTC	LCA30	9	74.13	72.28	75.97	70.15	78.30
Hip	CON13	7	19.85	19.02	20.69	18.72	21.48
Hip	CON30	8	18.33	17.89	18.77	17.22	19.04
Hip	LCA13	7	19.26	18.73	19.79	18.22	20.39
Hip	LCA30	9	19.05	18.45	19.66	17.75	20.42
Hyp	CON13	7	10.02	9.63	10.41	9.20	10.71
Hyp	CON30	8	10.12	9.90	10.33	9.67	10.51
Hyp	LCA13	7	9.95	9.59	10.31	9.20	10.51
Hyp	LCA30	9	10.33	9.88	10.78	9.32	11.53
Mid	CON13	7	11.60	11.31	11.89	11.05	12.07
Mid	CON30	8	11.84	11.56	12.12	11.39	12.41
Mid	LCA13	7	11.34	10.88	11.80	10.25	11.91
Mid	LCA30	9	12.22	11.81	12.62	11.30	13.01

Supplementary Table 11: Parameter: Absolute volume (continued from 10) of ROIs provided in mm^3. Amy = amygdala, hip = hippocampus, tha = thalamus, hyp = hypothalamus, BF = basal forebrain, EC = entorhinal cortex, OC = occipital cortex, mid = midbrain, pon = pons, FC = frontal cortex, PTC = parieto-temporal cortex. Lower = lower 95% CI, upper = upper 95% CI.

ROI	Group	n	Mean	Lower	Upper	Min	Max
Pon	CON13	7	14.53	13.97	15.08	13.34	15.38
Pon	CON30	8	14.61	14.25	14.97	13.77	15.23
Pon	LCA13	7	14.02	13.68	14.35	13.28	14.65
Pon	LCA30	9	15.13	14.68	15.58	14.08	16.15
Tha	CON13	7	15.73	15.22	16.24	14.89	16.73
Tha	CON30	8	15.11	14.81	15.41	14.35	15.65

ROI	Group	n	Mean	Lower	Upper	Min	Max
Tha	LCA13	7	15.40	15.08	15.72	14.78	15.87
Tha	LCA30	9	15.62	15.13	16.10	14.56	16.77
TBV	CON13	7	444.79	434.40	455.18	423.44	460.62
TBV	CON30	8	425.91	420.44	431.38	412.14	436.95
TBV	LCA13	7	433.97	424.19	443.74	407.24	446.93
TBV	LCA30	9	437.44	427.74	447.15	412.61	457.32

Supplementary Table 12: Parameter: Mean cortex thickness of ROIs provided in mm. FC = frontal cortex, PTC = parieto-temporal cortex, OC = occipital cortex, EC = entorhinal cortex, WC = whole cortex. Lower = lower 95% CI, upper = upper 95% CI.

ROI	Group	n	Mean	Lower	Upper	Min	Max
EC	CON13	7	0.69	0.68	0.70	0.68	0.71
EC	CON30	8	0.63	0.60	0.66	0.54	0.67
EC	LCA13	7	0.67	0.65	0.68	0.63	0.69
EC	LCA30	9	0.64	0.61	0.66	0.58	0.68
FC	CON13	7	1.08	1.06	1.11	1.02	1.12
FC	CON30	8	1.02	0.99	1.05	0.97	1.07
FC	LCA13	7	1.08	1.06	1.10	1.02	1.11
FC	LCA30	9	1.03	1.00	1.07	0.95	1.12
OC	CON13	7	0.83	0.82	0.85	0.81	0.87
OC	CON30	8	0.76	0.74	0.78	0.73	0.83
OC	LCA13	7	0.79	0.77	0.81	0.75	0.83
OC	LCA30	9	0.72	0.70	0.74	0.66	0.76
PTC	CON13	7	1.03	1.01	1.04	1.01	1.06
PTC	CON30	8	0.96	0.95	0.98	0.93	0.99
PTC	LCA13	7	1.01	1.00	1.03	1.00	1.05
PTC	LCA30	9	0.96	0.94	0.97	0.92	0.99
WC	CON13	7	0.97	0.96	0.98	0.94	0.99
WC	CON30	8	0.90	0.89	0.92	0.87	0.94
WC	LCA13	7	0.96	0.94	0.97	0.92	0.98
WC	LCA30	9	0.90	0.89	0.92	0.86	0.94

Supplementary Table 13: Parameter: MKT. Amy = amygdala, BFS = basal forebrain septum, hip = hippocampus, hyp = hypothalamus, IC = inferior colliculi, neo = neocortex, mid = midbrain, tha = thalamus. Lower = lower 95% CI, upper = upper 95% CI.

ROI	Group	n	Mean	Lower	Upper	Min	Max
Amy	CON13	7	0.627	0.589	0.664	0.561	0.691
Amy	CON30	8	0.536	0.516	0.556	0.496	0.577
Amy	LCA13	7	0.624	0.601	0.647	0.576	0.665
Amy	LCA30	8	0.511	0.479	0.544	0.430	0.581
BFS	CON13	7	0.658	0.626	0.689	0.570	0.691
BFS	CON30	8	0.587	0.557	0.616	0.523	0.672
BFS	LCA13	7	0.635	0.599	0.670	0.551	0.694

ROI	Group	n	Mean	Lower	Upper	Min	Max
BFS	LCA30	8	0.566	0.501	0.631	0.377	0.684
Hip	CON13	7	0.586	0.580	0.593	0.574	0.601
Hip	CON30	8	0.458	0.436	0.480	0.415	0.503
Hip	LCA13	7	0.568	0.547	0.589	0.537	0.606
Hip	LCA30	8	0.458	0.435	0.482	0.383	0.503
Hyp	CON13	7	0.919	0.877	0.962	0.817	0.974
Hyp	CON30	8	0.834	0.793	0.874	0.756	0.912
Hyp	LCA13	7	0.906	0.842	0.970	0.803	1.063
Hyp	LCA30	8	0.803	0.713	0.894	0.576	0.991
IC	CON13	7	0.887	0.837	0.937	0.768	0.951
IC	CON30	8	0.758	0.732	0.784	0.686	0.796
IC	LCA13	7	0.831	0.769	0.893	0.661	0.903
IC	LCA30	8	0.743	0.700	0.787	0.649	0.821
Neo	CON13	7	0.529	0.520	0.538	0.508	0.546
Neo	CON30	8	0.463	0.446	0.480	0.423	0.500
Neo	LCA13	7	0.524	0.511	0.537	0.494	0.552
Neo	LCA30	8	0.455	0.430	0.479	0.376	0.494
Mid	CON13	7	1.019	0.974	1.064	0.900	1.104
Mid	CON30	8	0.899	0.854	0.944	0.821	0.996
Mid	LCA13	7	0.947	0.880	1.013	0.788	1.056
Mid	LCA30	8	0.869	0.790	0.947	0.669	1.054
Tha	CON13	7	0.775	0.741	0.808	0.717	0.840
Tha	CON30	8	0.602	0.567	0.637	0.560	0.685
Tha	LCA13	7	0.740	0.688	0.792	0.673	0.859
Tha	LCA30	8	0.595	0.521	0.669	0.384	0.751

Supplementary Table 14: Parameter: MD. Amy = amygdala, BFS = basal forebrain septum, hip = hippocampus, hyp = hypothalamus, IC = inferior colliculi, neo = neocortex, mid = midbrain, tha = thalamus. Lower = lower 95% CI, upper = upper 95% CI.

ROI	Group	n	Mean	Lower	Upper	Min	Max
Amy	CON13	7	0.475	0.466	0.485	0.451	0.491
Amy	CON30	8	0.493	0.487	0.499	0.483	0.508
Amy	LCA13	7	0.477	0.472	0.482	0.467	0.484
Amy	LCA30	8	0.491	0.486	0.496	0.478	0.500
BFS	CON13	7	0.419	0.408	0.429	0.389	0.433
BFS	CON30	8	0.437	0.429	0.444	0.420	0.454
BFS	LCA13	7	0.420	0.411	0.429	0.401	0.431
BFS	LCA30	8	0.434	0.427	0.441	0.422	0.451
Hip	CON13	7	0.447	0.440	0.454	0.428	0.457
Hip	CON30	8	0.462	0.457	0.468	0.454	0.476
Hip	LCA13	7	0.447	0.441	0.453	0.436	0.458
Hip	LCA30	8	0.463	0.459	0.467	0.455	0.474
Hyp	CON13	7	0.391	0.382	0.401	0.365	0.404
Hyp	CON30	8	0.411	0.404	0.417	0.398	0.423
Hyp	LCA13	7	0.392	0.386	0.398	0.377	0.400
Hyp	LCA30	8	0.412	0.404	0.420	0.392	0.428
IC	CON13	7	0.394	0.384	0.404	0.363	0.404

ROI	Group	n	Mean	Lower	Upper	Min	Max
IC	CON30	8	0.418	0.411	0.424	0.399	0.427
IC	LCA13	7	0.394	0.387	0.401	0.380	0.409
IC	LCA30	8	0.418	0.413	0.423	0.404	0.426
Neo	CON13	7	0.458	0.449	0.466	0.437	0.469
Neo	CON30	8	0.470	0.463	0.476	0.456	0.483
Neo	LCA13	7	0.460	0.454	0.466	0.446	0.468
Neo	LCA30	8	0.471	0.467	0.476	0.465	0.483
Mid	CON13	7	0.379	0.369	0.389	0.352	0.392
Mid	CON30	8	0.403	0.395	0.411	0.381	0.416
Mid	LCA13	7	0.376	0.368	0.384	0.358	0.389
Mid	LCA30	8	0.402	0.398	0.407	0.388	0.410
Tha	CON13	7	0.391	0.381	0.400	0.367	0.404
Tha	CON30	8	0.405	0.398	0.412	0.391	0.419
Tha	LCA13	7	0.388	0.381	0.395	0.372	0.398
Tha	LCA30	8	0.405	0.399	0.410	0.387	0.412

Supplementary Table 15: Parameter: FA. Amy = amygdala, BFS = basal forebrain septum, hip = hippocampus, hyp = hypothalamus, IC = inferior colliculi, neo = neocortex, mid = midbrain, tha = thalamus. Lower = lower 95% CI, upper = upper 95% CI.

ROI	Group	n	Mean	Lower	Upper	Min	Max
Amy	CON13	7	0.309	0.298	0.320	0.278	0.323
Amy	CON30	8	0.272	0.258	0.286	0.229	0.291
Amy	LCA13	7	0.311	0.292	0.330	0.274	0.340
Amy	LCA30	8	0.271	0.257	0.285	0.247	0.309
BFS	CON13	7	0.346	0.335	0.357	0.314	0.358
BFS	CON30	8	0.325	0.312	0.338	0.292	0.352
BFS	LCA13	7	0.349	0.338	0.361	0.322	0.366
BFS	LCA30	8	0.319	0.305	0.332	0.293	0.348
Hip	CON13	7	0.311	0.300	0.322	0.292	0.332
Hip	CON30	8	0.258	0.253	0.264	0.245	0.268
Hip	LCA13	7	0.310	0.301	0.318	0.296	0.332
Hip	LCA30	8	0.264	0.259	0.270	0.255	0.275
Hyp	CON13	7	0.340	0.318	0.363	0.281	0.372
Hyp	CON30	8	0.328	0.298	0.358	0.258	0.372
Hyp	LCA13	7	0.339	0.318	0.361	0.292	0.375
Hyp	LCA30	8	0.331	0.306	0.356	0.269	0.382
IC	CON13	7	0.273	0.265	0.280	0.261	0.292
IC	CON30	8	0.263	0.259	0.268	0.254	0.273
IC	LCA13	7	0.274	0.265	0.283	0.258	0.294
IC	LCA30	8	0.261	0.253	0.268	0.240	0.275
Neo	CON13	7	0.267	0.260	0.274	0.253	0.279
Neo	CON30	8	0.229	0.225	0.233	0.219	0.239
Neo	LCA13	7	0.272	0.265	0.279	0.258	0.287
Neo	LCA30	8	0.229	0.222	0.237	0.217	0.247
Mid	CON13	7	0.381	0.365	0.397	0.357	0.409
Mid	CON30	8	0.337	0.322	0.352	0.299	0.363

ROI	Group	n	Mean	Lower	Upper	Min	Max
Mid	LCA13	7	0.381	0.361	0.400	0.356	0.431
Mid	LCA30	8	0.341	0.333	0.350	0.320	0.361
Tha	CON13	7	0.360	0.346	0.374	0.325	0.384
Tha	CON30	8	0.324	0.315	0.332	0.295	0.334
Tha	LCA13	7	0.363	0.351	0.375	0.341	0.394
Tha	LCA30	8	0.327	0.321	0.333	0.317	0.342
